# VapC Toxins from *Mycobacterium tuberculosis* Are Ribonucleases that Differentially Inhibit Growth and Are Neutralized by Cognate VapB Antitoxins

**DOI:** 10.1371/journal.pone.0021738

**Published:** 2011-06-29

**Authors:** Bintou Ahmadou Ahidjo, Diane Kuhnert, Joanna L. McKenzie, Edith E. Machowski, Bhavna G. Gordhan, Vickery Arcus, Garth L. Abrahams, Valerie Mizrahi

**Affiliations:** 1 Medical Research Council, National Health Laboratory Service, University of the Witwatersrand Molecular Mycobacteriology Research Unit, Department of Science and Technology, National Research Foundation Centre of Excellence for Biomedical Tuberculosis Research, Faculty of Health Sciences, University of the Witwatersrand and the National Health Laboratory Service, Johannesburg, South Africa; 2 Department of Biological Sciences, University of Waikato, Hamilton, New Zealand; University of Hyderabad, India

## Abstract

The chromosome of *Mycobacterium tuberculosis* (Mtb) encodes forty seven toxin-antitoxin modules belonging to the VapBC family. The role of these modules in the physiology of Mtb and the function(s) served by their expansion are unknown. We investigated ten *vapBC* modules from Mtb and the single *vapBC* from *M. smegmatis*. Of the Mtb *vapC*s assessed, only *Rv0549c*, *Rv0595c*, *Rv2549c* and *Rv2829c* were toxic when expressed from a tetracycline-regulated promoter in *M. smegmatis*. The same genes displayed toxicity when conditionally expressed in Mtb. Toxicity of Rv2549c in *M. smegmatis* correlated with the level of protein expressed, suggesting that the VapC level must exceed a threshold for toxicity to be observed. In addition, the level of Rv2456 protein induced in *M. smegmatis* was markedly lower than Rv2549c, which may account for the lack of toxicity of this and other VapCs scored as ‘non-toxic’. The growth inhibitory effects of toxic VapCs were neutralized by expression of the cognate VapB as part of a *vapBC* operon or from a different chromosomal locus, while that of non-cognate antitoxins did not. These results demonstrated a specificity of interaction between VapCs and their cognate VapBs, a finding corroborated by yeast two-hybrid analyses. Deletion of selected *vapC* or *vapBC* genes did not affect mycobacterial growth *in vitro*, but rendered the organisms more susceptible to growth inhibition following toxic VapC expression. However, toxicity of ‘non-toxic’ VapCs was not unveiled in deletion mutant strains, even when the mutation eliminated the corresponding cognate VapB, presumably due to insufficient levels of VapC protein. Together with the ribonuclease (RNase) activity demonstrated for Rv0065 and Rv0617 – VapC proteins with similarity to Rv0549c and Rv3320c, respectively – these results suggest that the VapBC family potentially provides an abundant source of RNase activity in Mtb, which may profoundly impact the physiology of the organism.

## Introduction

The feature of Mtb that presents the most significant impediment to developing treatment-shortening therapies for tuberculosis (TB) is its remarkable ability to persist in the face of the threats imposed by host immunity and bactericidal drug action [1], [2], [3]. This ability is thought to account, at least in part, for the protracted duration of treatment required to cure TB with short-course chemotherapy. Thus, understanding the physiology of the persistent state/s in Mtb represents one of the most important areas of mycobacterial research today [1], [3], [4]. Much emphasis has been placed on defining the physiology of non-replicating persister cells of Mtb formed under the conditions of nutritional, hypoxic, nitrosative and acidic stress thought to be encountered during infection [5], [6]. This has led to the identification of pathways that are required to maintain mycobacterial viability under such conditions and whose components are being pursued as novel drug targets [7], [8], [9], [10], [11]. Attention has also focused on the mechanisms underlying the formation of persister cells that arise through the stochastic expression of proteins that can affect the physiology and growth rate of the cell by interfering with key cellular processes such as macromolecular synthesis [2], [12], [13], [14], [15], [16], [17], [18], [19], [20]. These mechanisms are the subject of considerable interest having been implicated in phenotypic drug tolerance/indifference in *E. coli* [2], [14], [15], [16], [17], [18], [21], [22], [23].

Of the genes implicated in such processes, most attention has been paid to toxin-antitoxin (TA) modules, which are bicistronic operons widely distributed in the genomes of free-living prokaryotes [24], [25]. Although their contemporary role in microbial physiology remains the subject of debate [26], [27], there is evidence that chromosomal TA modules may act in stress physiology by serving as metabolic regulators of growth [13], [25], [28], [29]. When bound in a complex, the antitoxin neutralizes the activity of the toxin [30], [31], [32]. In the absence of continued expression of the operon, which regulates its own expression [33], dissociation of the complex and degradation of the relatively unstable antitoxin unveils the biological activity of the toxin.

Mtb possesses an unusually large and diverse complement of TA modules which belong to the MazEF, RelBE, ParDE, HigBA and VapBC families [24], [25]. A systematic analysis of Mtb TA module function revealed that Mtb also possesses a number of novel systems with no similarity to known modules [34]. Within this repertoire, the paralogous expansion of the VapBC family is particularly noteworthy [24], [25], [35], [36] and is a feature that Mtb shares with a small number of unrelated organisms [37]. In stark contrast, the genomes of mycobacteria other than those belonging to the Mtb complex (*M. africanum*, *M. microti*, *M. canetti* and *M. bovis*) are virtually devoid of VapBC and other TA modules [34]. The TA modules of Mtb have been the subject of intense investigation [31], [34], [38], [39], [40], [41], [42], [43], [44], [45]. Of the 47 VapBC modules (Fig. S1), one has been structurally and biochemically characterized [31], and this and 22 others tested for toxicity in *E. coli* [40], [45]. Twenty one of 45 VapCs tested were found to be toxic in *M. smegmatis* and four of these were shown to inhibit translation [34]. VapC toxins belong to the PIN (PilT N-terminus) domain family of proteins whose members have been associated with nuclease activity [36]. Most recently, enteric VapCs were shown to act as site-specific endonucleases that inhibit translation by cleavage of initiator tRNA [46]. PIN domains have RNase-H-like fold in which four conserved acidic residues are located in close proximity to form a negatively charged pocket, as illustrated in the structures of the VapC from *Pyrobaculum aerophilum* [47], [48], VapC5 from Mtb [31] and FitB from *Neisseria gonorrhoeae* [32]. Dissociation of the toxin-antitoxin complex is thought to allow binding of divalent metal ion in this acidic pocket of VapC thereby creating an active site for metal-ion-dependent nuclease activity [32], [48].

To investigate *vapBC* function in mycobacteria, we focused on a subset of 10 modules from Mtb H37Rv and the single *vapBC* from *M. smegmatis* mc^2^155. We found that some, but not all of the VapC proteins confer growth inhibition following inducible over-expression in both mycobacterial species. The toxic activity of these VapCs could be neutralized by the cognate, but not non-cognate antitoxins, indicating that these loci encode functional VapBC modules. A correlation between the expression levels of the VapC proteins and its ability to confer a toxic phenotype was observed. Analysis of mycobacterial *vapBC* deletion mutants failed to yield observable phenotypes under standard growth conditions. However, toxic VapCs showed enhanced toxicity when expressed in deletion mutants lacking antitoxic VapCs, providing further evidence of the specificity of interaction between VapCs and their cognate antitoxins, as revealed by yeast two-hybrid analyses of VapB-VapC interactions. Finally, the VapCs, Rv0065 and Rv0617, which share ∼50% sequence similarity to the toxic VapCs, Rv0549c and Rv3320c, respectively, were shown to have sequence-selective, Mg^2+^-dependent RNase activity, further confirming an association between VapC toxicity, translational inhibition and RNA cleavage. We discuss the implications of these findings for the physiology of Mtb.

## Results

### VapBC modules selected for study

A subset of VapBC modules in Mtb was selected for study with the choice being guided, in part, by information on transcriptional responsiveness and/or essentiality of *vapBC* gene function available at the time (Table S1). The selected modules are also widely distributed among the main branches of the Mtb VapC phylogenetic tree (Fig. S2). Certain *vapB*-encoded antitoxins were reported to be induced during infection of human macrophages (Rv0550c, Rv2009, Rv2547 and Rv3321c) [34], [49]. The toxin Rv0627 and antitoxin Rv2830c were identified as essential for growth in vitro [50], with the latter induced by hypoxia [51]. The cluster of three *vapBC* modules located contiguously on the chromosome (*Rv2545-Rv2546*, *Rv2547-Rv2548* and *Rv2550c-Rv2549c*) was selected based on the transcriptional responsiveness of most of the genes under in-vivo-relevant conditions [52], [53], [54], [55], [56], [57] and the requirement of Rv2548 for intracellular fitness [58]. The C-terminally truncated Rv1953 was included as a negative control as it lacks residues comprising part of the PIN domain and, as such, is predicted to lack nuclease activity. In addition to the ten Mtb modules, we also included the *M. smegmatis vapBC*, *MSMEG_1283-MSMEG_1284*, in our analysis. During the course this study, new information on these modules became available from other studies [31], [34], [40], [59].

### Differential growth inhibitory effects of VapC toxins in *M. smegmatis*

**Figure 1:**
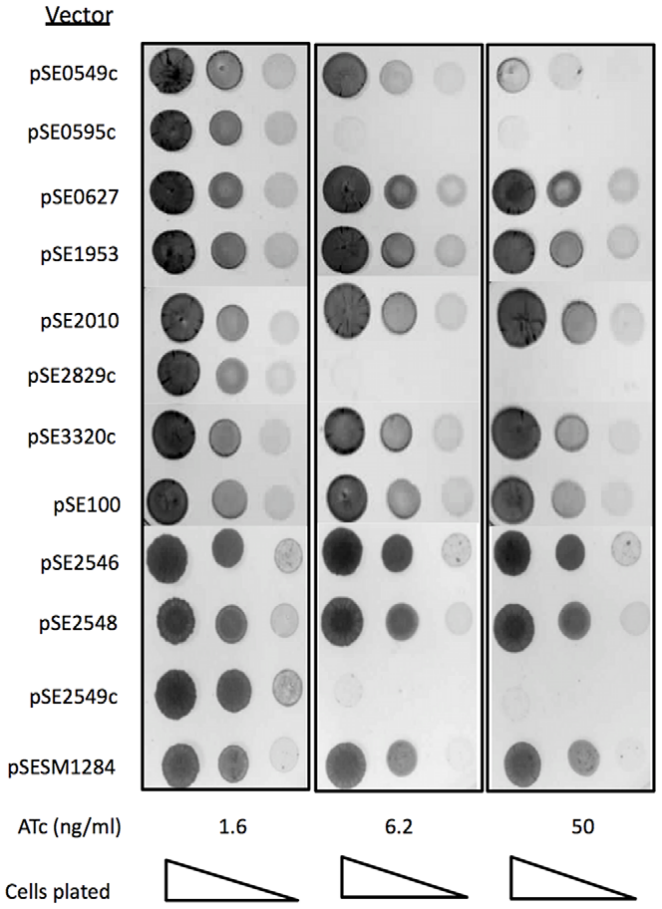
Effect of mycobacterial VapC expression on growth of *M. smegmatis* on solid media. Ten-fold serial dilutions of cells were spotted on 7H10 agar without or with ATc (1.6, 6.2 and 50 ng/ml) and incubated for 24–48 h.

VapC toxicity was initially assessed in *M. smegmatis* by conditional expression of their encoding genes using an uncoupled system in which the toxin was expressed from a tetracycline (Tet)-regulated promoter (P_myc1_ *tetO)* contained on the episomal plasmid, pSE100 [60]. In this system, repression from the P_myc1_ *tetO* promoter in the absence of Tet inducer was mediated by the wild type Tet-repressor (TetR) constitutively expressed from the strong P_smyc_ promoter contained on an L5-based integration vector (pMC1s) [60]. The resulting vector pairs were co-electroporated into *M. smegmatis*, and in all cases, were found to be stable in the absence of anhydrotetracycline (ATc) inducer, suggesting that *vapC* expression was sufficiently repressed to avoid plasmid loss or mutation as a result of toxic gene expression. The toxicity of the VapC proteins was assessed by spotting dilutions of cultures on solid media containing ATc at a concentration of 0–50 ng/ml. Of the ten VapCs assessed, only four, namely, Rv0549c, Rv0595c, Rv2549c and Rv2829c, were growth inhibitory in *M. smegmatis* at ATc concentrations >3 ng/ml; the toxic effect of Rv0549c expression was, however, noticeably less than that of the other VapCs (Fig. 1). No toxicity was observed following induction of the others VapC, including MSMEG_1284, with ATc at concentrations up to 200 ng/ml.

**Figure 2:**
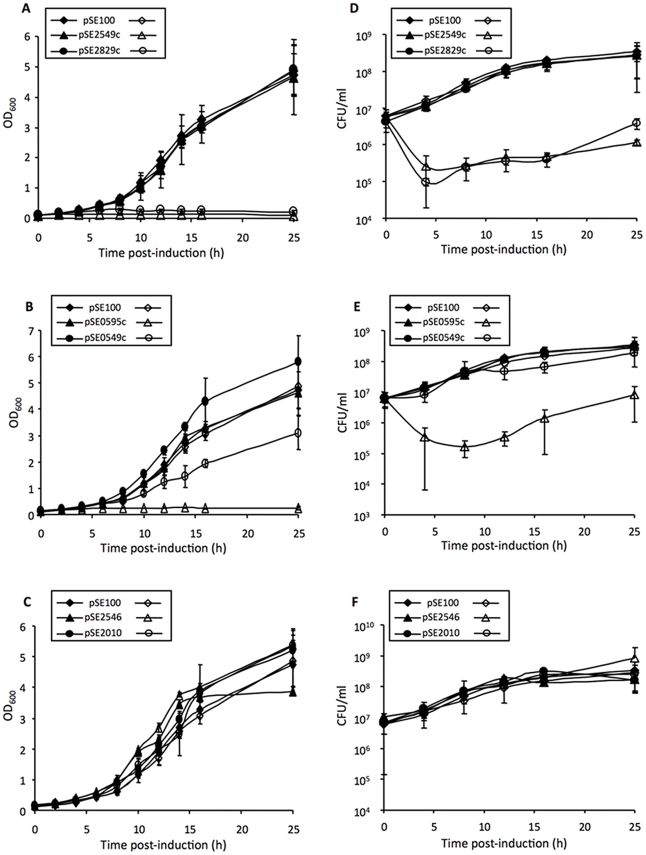
Variable effects of Mtb VapC over-expression on growth and viability of *M. smegmatis* in liquid culture. Growth and viability were assessed spectrophotometrically (**A, B, C**) and by CFU enumeration (**D, E, F**). Open symbols represent ATc-induced samples and filled symbols represent uninduced controls. The results represent the average and standard deviations from one of three independent experiments.

The effect of ectopic VapC expression on the viability of *M. smegmatis* was then assessed by inducing toxin expression during growth in liquid media (Fig. 2). For three VapCs that were inhibitory in the spotting assay (Rv0595c, Rv2549c and Rv2829c), *vapC* induction resulted in a ca. 2-log_10_ reduction in CFUs after 3 h of induction; thereafter, the CFU values stabilized before increasing again between 10 and 24 h post-induction (Fig. 2, panels D and E). Six randomly selected colonies from the Rv2829c-expressing strain that grew on plates after 16 h exposure to ATc in liquid culture were picked and phenotypically analyzed alongside colonies recovered from an ATc-free control. All colonies from the control were severely growth-impaired when plated on ATc confirming that inducible VapC expression was retained in these cells. In contrast, four clones derived from the ATc-induced sample were no longer resistant to hygromycin (Hyg) or had lost responsiveness to ATc. Similar results were obtained in the case of Rv0595c and Rv2549c: all colonies recovered after prolonged ATc exposure had undergone plasmid loss or rearrangement with concomitant loss of ATc responsiveness of growth (data not shown). Therefore, high-level expression of toxic VapCs exerts a selective pressure that promotes plasmid loss or instability in *M. smegmatis*.

Since use of a strong promoter to drive expression of *tetR* may limit the ability to de-repress *vapC* expression by ATc treatment and thus mask the activity of VapCs for which no growth inhibition was observed, we replaced pMC1s with pMC2m, in which *tetR* is expressed from an intermediate strength promoter. However, in this configuration, no toxicity was observed for Rv0627, Rv1953, Rv2010, Rv2546, Rv2548, Rv3320c and MSMEG_1284, even at the highest concentration of ATc tested (200 ng/ml) (data not shown). The effect of fully de-repressing *vapC* expression was then assessed by measuring the transformation efficiencies in *M. smegmatis* of the expression vectors in the absence of TetR. The transformation efficiencies of pSE0549c, pSE0595c, pSE2549c, pSE2829c and pSE3320c were ≥3-log_10_ lower than the empty vector control, pSE100 (Table S2). Of these, four were previously scored as toxic when conditionally expressed (Rv0549c, Rv0595c, Rv2549c and Rv2829c), whereas toxicity of Rv3320c was only revealed when this VapC was constitutively expressed. In contrast, the transformation efficiencies of pSE0627, pSE1953, pSE2010, pSE2546, pSE2548 and pSESM1284 were comparable to that of pSE100 (0.08-1.3-fold), confirming that no significant toxicity was conferred by these VapCs, even when constitutively expressed (Table S2). We tested whether Rv2546, Rv2548 and MSMEG_1284 may have been rendered non-toxic through plasmid mutation/rearrangement by sequencing the promoter-operator region and insert of plasmid recovered from transformants obtained with and without the *tetR*-expressing vector. However, in all cases, the recovered plasmid was unaltered (data not shown).

### Differential growth inhibitory effects of VapC toxins in wild type Mtb

**Figure 3:**
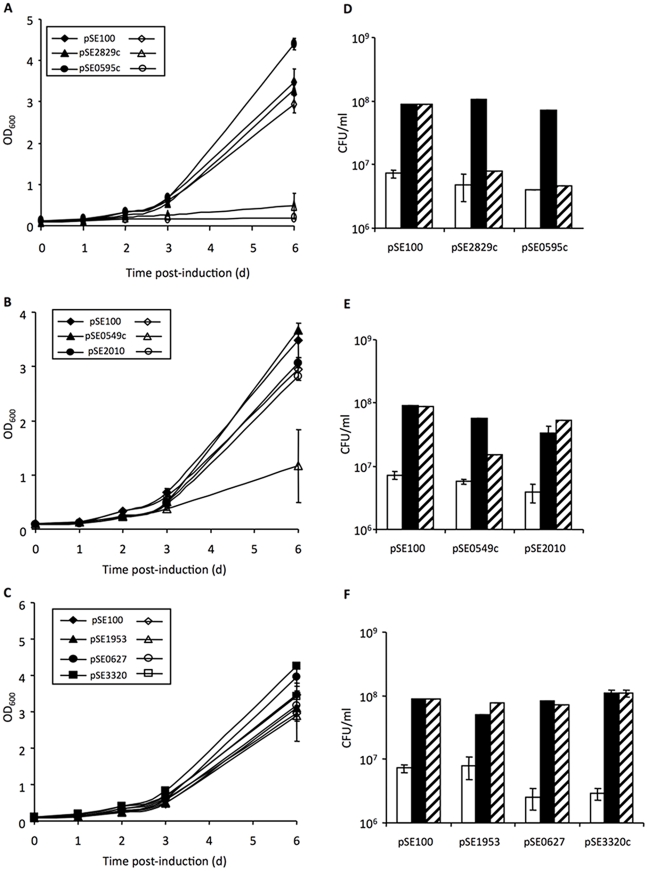
Variable effects of Mtb VapC expression on growth and viability of wild type Mtb in liquid culture. **A, B, C:** Growth was assessed spectrophotometrically for a period of 6 days after induction of gene expression, as described under Materials and Methods. Open symbols represent ATc-induced samples and filled symbols represent uninduced controls. The results show the data from one of three independent experiments. **D, E, F:** Viability was assessed by enumerating CFU. Open bars, CFU prior to induction of gene expression (day 0); black bars, untreated control (day 3); and striped bars, ATc-treated culture (day 3). The results show the average CFU values from duplicate platings at one serial dilution from one of three independent experiments.

**Figure 4:**
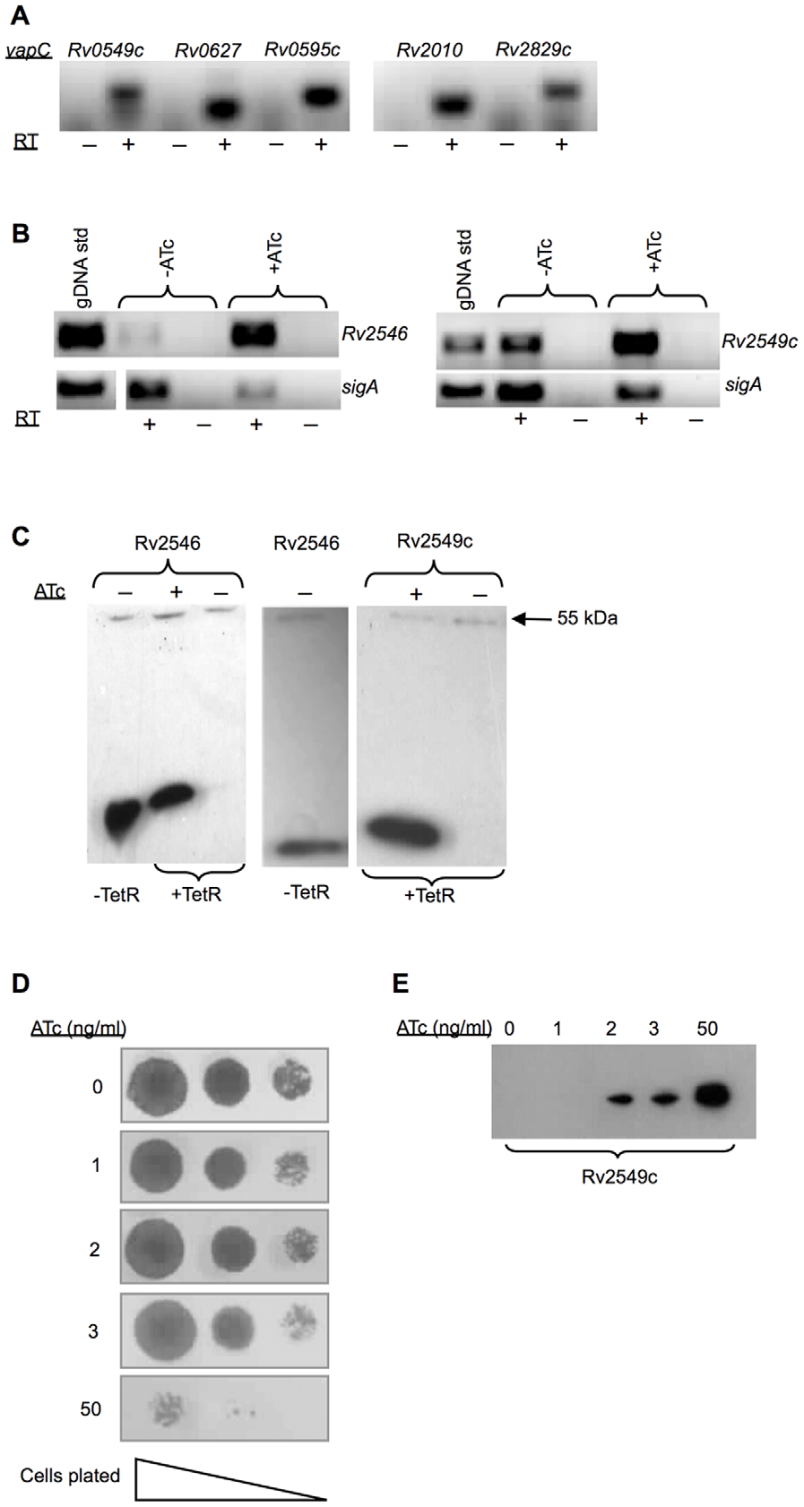
Analysis of ATc-regulated *vapC* expression in mycobacteria. Expression was analyzed by RT-PCR (**A** and **B**) and detection of expressed proteins by epitope tagging (**C**). RT-PCR was carried out using mRNA samples obtained from (**A**) induced cultures of wild type Mtb (6 h treatment with ATc at 25 ng/ml); and (**B**) ATc-induced and uninduced cultures of *M. smegmatis* (1 h treatment with ATc at 50 ng/ml) over-expressing various *vapC* toxins under the control of the P_myc1_ *tetO* promoter. Samples obtained with (+) and without (-) reverse transcription (RT) were compared to distinguish cDNA from genomic DNA contamination. (**C**) Cellular fractions isolated from *M. smegmatis* were subjected to Western blot analysis using the anti-FLAG M2 antibody to detect the epitope-tagged VapC Rv2546 and Rv2549c proteins. Cultures were grown in either the presence (+) or absence (-) of 50 ng/ml ATc for 3 h. (**D**) The effect of epitope tagged Rv2549c expression on the growth of *M. smegmatis* on solid media. Ten-fold serial dilutions of cells were spotted on 7H10 agar without or with the ATc (1, 2, 3 and 50 ng/ml) and incubated for 24–48 h. (**E**) Comparison of the relative abundance of Rv2549c induced with varying concentrations of ATc. Cultures were grown in either the presence (+) or absence (−) of 50 ng/ml ATc for 3 h. Equal amounts (3 µg) were subjected to Western blot analysis using the anti-FLAG M2 antibody to detect the epitope-tagged Rv2549c protein.

The effect of regulated expression of a subset of VapCs was analyzed in wild type Mtb (Fig. 3 and data not shown). Rv0627, Rv1953, Rv2010, Rv2546, Rv2548 and Rv3320c had no effect on the growth of H37Rv in liquid culture (Fig. 3C, Fig. 3F and data not shown) even though corresponding *vapC* transcript was detected (Fig. 4A and data not shown). However, as observed in *M. smegmatis*, only Rv0549c, Rv0595c, Rv2549c and Rv2829c were growth inhibitory when conditionally expressed in Mtb, with Rv0549c being the least toxic (Fig. 3, panels A, B, D and E). Moreover, while expression of the toxic VapCs in *M. smegmatis* resulted in a marked decline in CFUs after ATc induction (Fig. 2), no reduction in CFUs was observed over a 3–4-day induction period in Mtb (Fig. 3D, Fig. 3E and data not shown) with CFUs remaining unchanged over this period. The effect of constitutive VapC expression in Mtb was then assessed by measuring the transformation efficiencies of the VapC expression vectors in the absence of a *tetR*-expressing partner (Table S3). Toxicity of Rv0549c, Rv0595c, Rv2549c and Rv2829c was revealed by reduced transformation efficiency relative to the empty vector control. Moreover, as in *M. smegmatis* (Table S2), toxicity of Rv3320c in Mtb was only revealed when this VapC was constitutively expressed (Table S3).

### Detection of VapC toxicity is influenced by the level of expressed protein

To investigate the reasons underlying the apparent lack of toxicity of some VapCs, transcript levels of the genes encoding the toxic Rv2549c and non-toxic Rv2546 VapCs were compared by RT-PCR analysis of mRNA from *M. smegmatis* cells cultured with or without ATc (Fig. 4B). Expression of both genes was significantly induced by ATc. Similar results were obtained for the other *vapC*s investigated in this study (data not shown) suggesting that the lack of toxicity was not due to a defect in gene expression from the Tet-regulated promoter. To compare the levels of VapC proteins induced in *M. smegmatis*, we tagged Rv2549c and Rv2546 at their C-termini with the 3×FLAG epitope. Epitope-tagged Rv2549c displayed toxicity equivalent to its native counterpart, confirming that the 3×FLAG tag did not affect the biological activity of this VapC (data not shown). Immuno-reactive bands of the size predicted for epitope-tagged Rv2546 and Rv2549c proteins (∼17 kDa) were observed in Western blots of cell-free extracts from *M. smegmatis* strains carrying the tagged VapC expression vectors that were cultured in the presence ATc (Fig. 4C, “+ATc”). A 55-kDa immuno-cross-reactive band detected in the cell-free extract of *M. smegmatis* provided an internal control that allowed the levels of FLAG-tagged VapCs to be compared between strains. The level of Rv2546 protein was noticeably higher in cells lacking TetR than in ATc-induced cells carrying TetR (Fig. 4C; left-hand panel, “-TetR” vs. “+TetR” lanes). However, the level of Rv2546 under fully de-repressed conditions was still lower than that of Rv2549c in TetR-containing cells induced with ATc (Fig. 4C, middle and left-hand panels). These results suggest that the level of Rv2546 might have been insufficient to cause a growth inhibitory effect in *M. smegmatis.*

To further investigate the effects of VapC levels on cell viability, we analysed the effect of epitope-tagged Rv2549c over-expression on the growth of *M. smegmatis* at various concentrations of ATc, and compared this with the level of protein expressed in cells cultured under the same conditions. As shown previously (Fig. 1), the toxic effect of Rv2549c expression only became evident at ATc concentrations >3ng/ml, whereas lower concentrations of inducer had little or no impact on the growth or viability of *M. smegmatis* (Fig. 4D). Western blot analysis of cell-free extracts of *M. smegmatis* cultures exposed to the same concentrations of inducer revealed an ATc dose-dependent increase in the level of epitope-tagged Rv2549c (Fig. 4E). Therefore, although Rv2549c is expressed at the lower concentrations of inducer (≤3 ng/ml), these results suggest that a specific threshold must be breached in order for the toxic activity of this VapC to become apparent in *M. smegmatis*.

### Abrogation of VapC toxicity by cognate antitoxin expression

**Figure 5:**
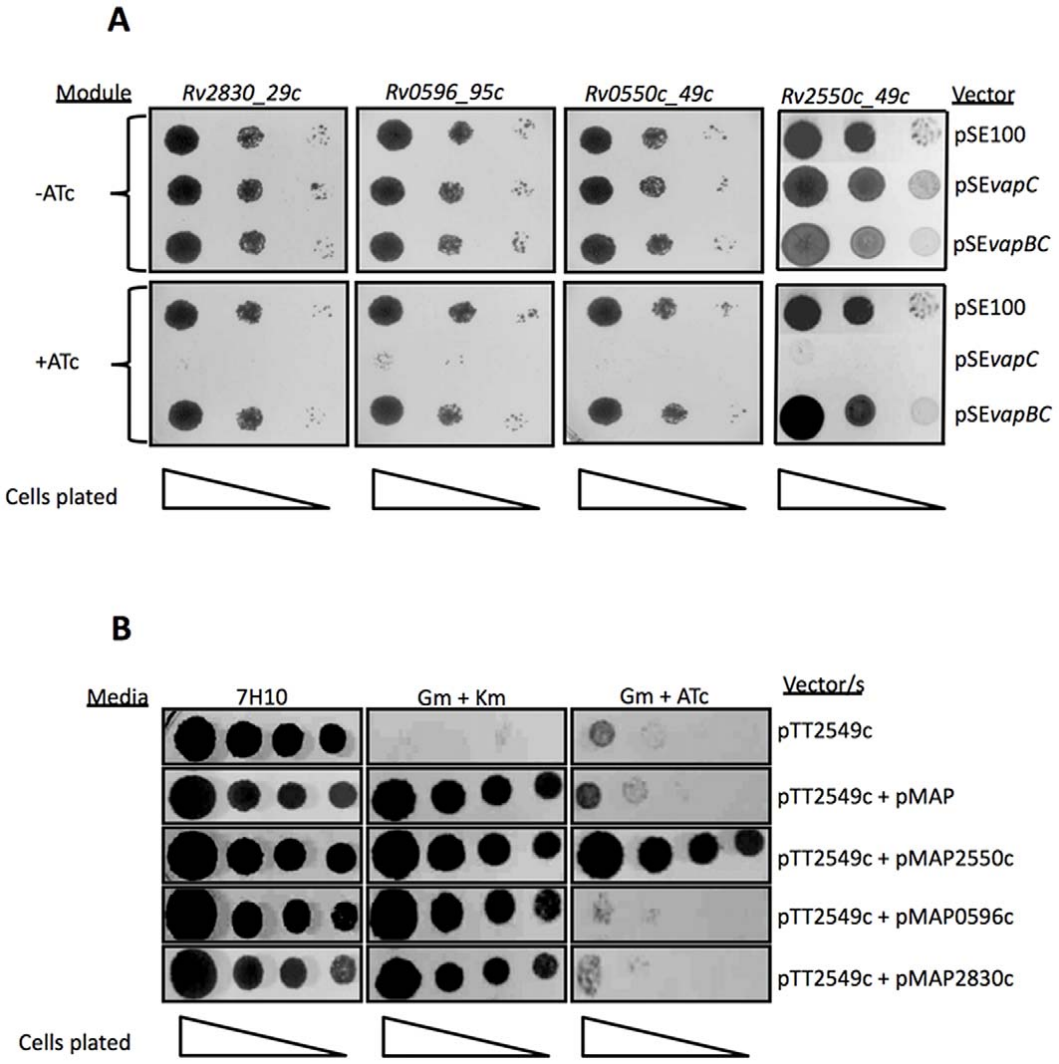
Neutralization of Mtb VapC toxicity in *M. smegmatis* by cognate antitoxin expression. **A:** Cognate toxin-antitoxin modules were co-expressed as an operon under control of the Tet-regulated promoter. Serial dilutions were plated on 7H10 agar alone (-ATc) or with ATc at 25 ng/ml (+ATc). **B:** Toxin and antitoxin genes were expressed separately on Tweety (*vapC*) or L5-based integration vectors (*vapB*) under control of Tet- or acetamide-regulated promoters, respectively. Serial dilutions were plated on 7H10 agar alone or supplemented with Gm and Km, or Gm and ATc (50 ng/ml).

To determine whether the activity of the toxic VapC proteins could be neutralized by expression of their cognate antitoxins, we compared the growth of *M. smegmatis* strains in which the *vapC* gene was expressed either individually from the P_myc1_ *tetO* promoter, or together with its cognate *vapB* gene in its native configuration as a *vapBC* operon. As observed previously (Fig. 1), ATc-induced expression of the VapCs encoded by *Rv0549c*, *Rv0595c*, *Rv2549c* and *Rv2829c* resulted in growth inhibition in the absence of its cognate antitoxin (Fig. 5A). However, in all cases, co-expression of the toxin together with its cognate antitoxin rescued the growth of the strains in the presence of ATc (Fig. 5A). The finding that VapC toxicity is abrogated by cognate VapB antitoxin co-expression supports the notion that these proteins form functional TA pairs in mycobacteria, and that the growth inhibition observed in the absence of antitoxin was not due to generalized toxicity due to protein over-expression.

We then investigated whether the ability to neutralize VapC activity is restricted to cognate antitoxins using an uncoupled system in which the genes encoding toxic VapCs were expressed from the ATc-inducible promoter on a Tweety-based integration vector [61] carrying *tetR*, and the antitoxins constitutively expressed from the acetamidase promoter [62] on a L5-based integration vector. In this configuration, growth inhibition of *M. smegmatis* following ATc-induced over-expression of the Rv2549c VapC protein was retained (Fig. 5B). The toxicity was, however, neutralized by co-expression of its cognate VapB, Rv2550c, when its encoding gene was integrated at a chromosomal locus distal from that of the toxin (Fig. 5B). In contrast, expression of the non-cognate antitoxins, Rv0596c and Rv2830c, which had previously been shown to abolish toxicity of their cognate toxins (Fig. 5A), had no effect on the growth inhibition caused by induction of Rv2549c (Fig. 5B). Similarly, expression of its cognate VapB, Rv0596c, alleviated the toxicity of Rv0595c in the presence of ATc whereas expression of Rv2550c or Rv2830c had no effect (data not shown). Together, these data confirm that the activity of VapC toxins can only be neutralized by their cognate antitoxins.

### Specificity of VapC interaction with cognate VapB confirmed by yeast-two hybrid (Y2H) analysis

**Figure 6:**
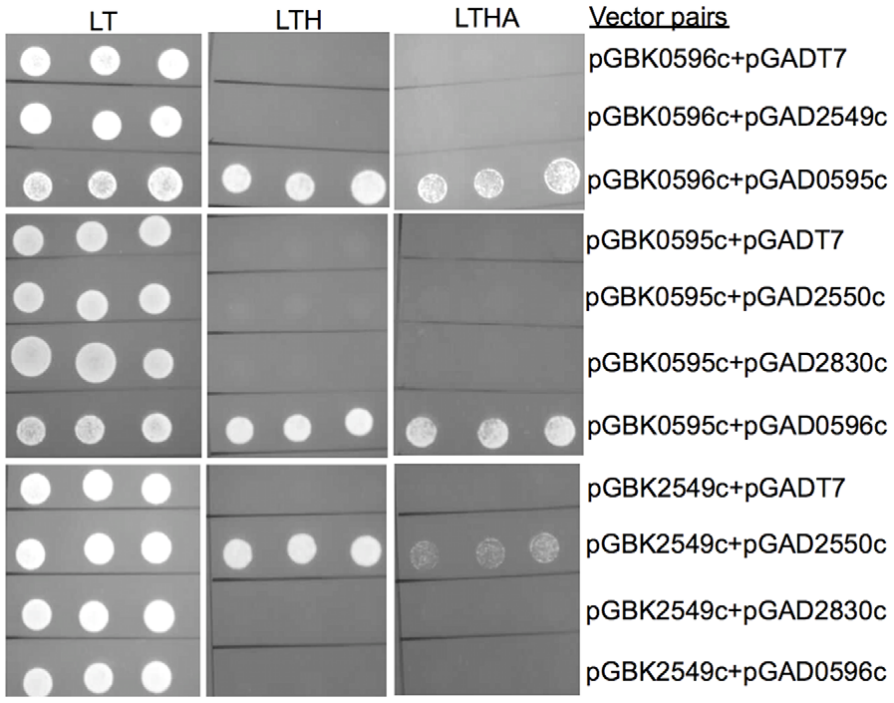
Y2H analysis of VapC-VapB interactions. Interactions between VapCs and either cognate or non-cognate VapBs were tested by co-transformation and scoring for growth on selection (LT) *vs*. media of differing stringency. Three independent colonies of each strain were re-suspended in sterile water, the cell density adjusted to an OD_600_ of 1 and aliquots spotted on plates. LT, Leu-Trp; LTH, Leu-Trp-His; LTHA, Leu-Trp-His-Ade dropout-supplemented media.

The interaction between cognate vs. non-cognate VapC and VapB proteins was further analyzed by Y2H analysis (Fig. 6). In this assay system, the VapC, Rv0595c, was found to interact specifically with its cognate VapB, Rv0596c as evidenced by growth of the corresponding yeast strains on high-stringency media. In contrast, no interaction was observed between Rv0595c and the non-cognate VapBs, Rv2550c and Rv2830c, or between Rv0596c and the non-cognate VapC, Rv2549c. Similar results were obtained for Rv2549c, which interacted specifically with Rv2550c, but showed no detectable interaction with the non-cognate VapBs, Rv2830c and Rv0596c. These results independently confirm the specificity of cognate VapB-VapC interaction deduced from the toxicity neutralization data and suggest that spurious interactions between non-cognate VapB-VapC pairs may not be functionally relevant in the native host.

### Effect of *vapBC* loss on susceptibility to cognate *vapC*-mediated toxicity

To investigate the role of the *vapBC* TA modules in the physiology of mycobacteria, unmarked Mtb deletion mutants lacking the *vapC*, *Rv0595c*, the *vapBC, Rv2009-Rv2010*, or the three contiguous *vapBC* modules, *Rv2545-Rv2546*, *Rv2547-Rv2548* and *Rv2550c-Rv2549c*, were generated by allelic exchange mutagenesis. In addition, a *M. smegmatis* mutant lacking the *MSMEG_1283-MSMEG_1284* module was constructed. None of the strains displayed a growth defect relative to the wild type when cultured under standard conditions in either liquid medium or on agar plates (data not shown). The susceptibility of the *M. smegmatis vapBC* mutant to cell wall, oxidative, nitrosative, genotoxic and heat stress was also assessed and found to be indistinguishable from wild type under all conditions tested (data not shown).

**Figure 7:**
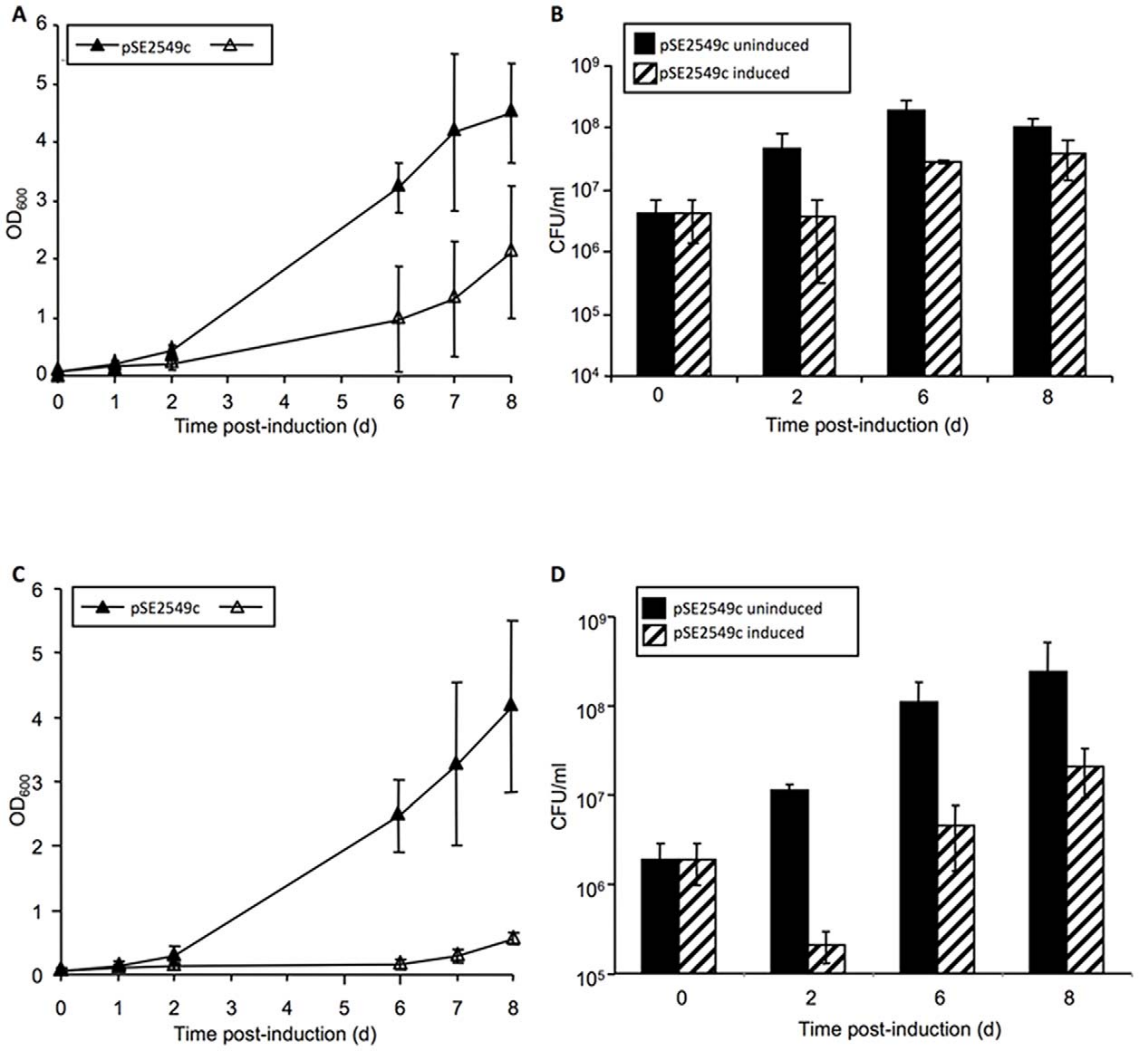
Rv2549c displays increased toxicity in the Δ*Rv2545-Rv2550c* mutant strain of Mtb. Growth and viability were assessed spectrophotometrically (**A, C**) and by CFU enumeration (**B, D**). Open symbols represent ATc-induced samples and filled symbols represent uninduced controls. The results represent the average and standard deviations from three independent experiments.

To assess the effect of loss of *vapB* function on the susceptibility of Mtb to growth inhibition by cognate or non-cognate *vapC* expression, we compared the transformation efficiencies of the *vapC* expression vectors in the Δ*Rv2545-Rv2550c* mutant and wild type strains (Table S3). Neither *Rv2546* nor *Rv2548* was toxic when expressed in the mutant strain, which lacks their cognate *vapB* genes. Similarly, no toxicity was observed when *MSMEG_1284* was conditionally or constitutively expressed in wild type *M. smegmatis* or the deletion mutant lacking the *MSMEG_1283-MSMEG_1284* module. In contrast, an exacerbation of Rv2549c toxicity in the absence of its cognate antitoxin was revealed by comparing its effects on growth and viability in Mtb when conditionally expressed in liquid cultures of the wild type vs. Δ*Rv2545-Rv2550c* strains (Fig. 7). Expression of *Rv2549c* was growth inhibitory in wild type Mtb (Figs. 7A and B); however, a 10-fold reduction in CFUs was observed within two days of induction of *Rv2549c* expression in the mutant strain (Figs. 7C and D). As in *M. smegmatis*, the outgrowth observed subsequent to the early killing of the Δ*Rv2545-Rv2550c* mutant upon induction of *Rv2549c* expression was due to abrogation of toxicity by plasmid rearrangement or loss (data not shown).

### The VapCs Rv0065 and Rv0617 display sequence-selective RNase activity

**Figure 8:**
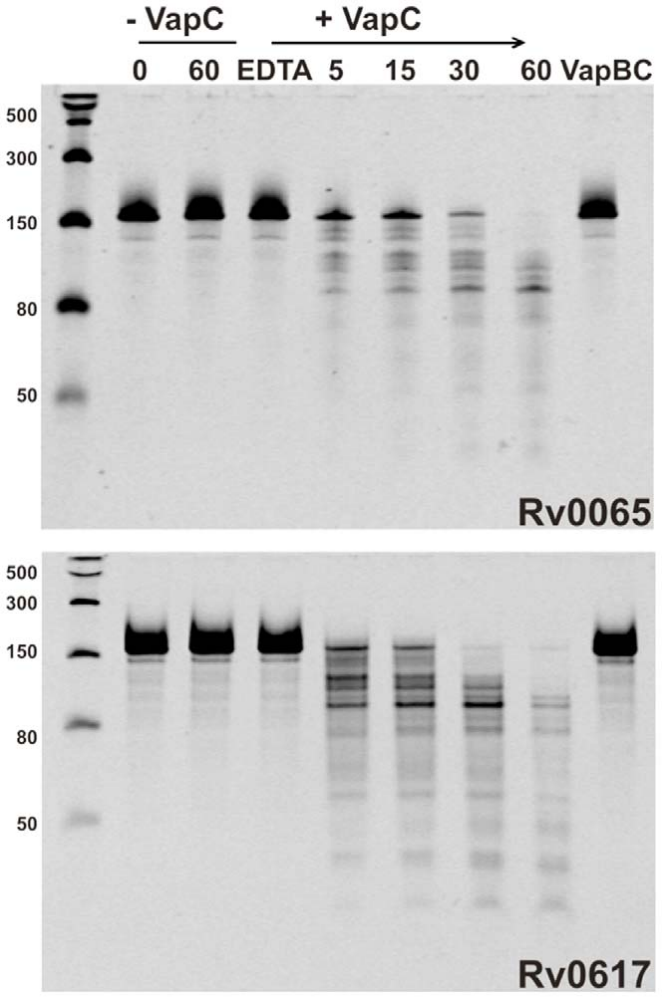
The VapCs Rv0065 and Rv0617 have RNase activity. VapC proteins Rv0065 and Rv0617 display sequence-selective RNase activity against an RNA substrate of ∼150 bases in the presence of 6 mM MgCl_2_. Activity is Mg^2+^-dependent as addition of 12 mM EDTA abolishes activity (lane 4). Ribonuclease activity is also inhibited in the presence of VapB (lane 9). Addition of VapC (+VapC) results in degradation of the RNA substrate over a period of 5 – 60 minutes (lanes 5–8). RNA only controls (-VapC) show no contaminating ribonuclease activity (lanes 2 & 3). The molecular weight marker shows molecular masses of single-stranded RNA in number of bases (lane 1).

To investigate the mechanism underlying VapC toxicity in mycobacterial hosts, we sought to biochemically characterize five VapCs analyzed in this study (Rv0549c, Rv0595c, Rv2546, Rv2549c and Rv2829c). In general, mycobacterial VapC proteins cannot be expressed in *E. coli* due to a combination of their insolubility and toxicity (J.L.M & V.A., unpublished observations). Thus, the VapCs were expressed in *M. smegmatis* [59], [63] as tagged fusion protein complexes with their cognate VapBs. The Rv0596c-Rv0595c and Rv2830c-Rv2829c complexes did not express in *M. smegmatis*, and the other three complexes did express but the proteins were insoluble after cell lysis (Rv0550c-Rv0549c, Rv2545-Rv2546 and Rv2550c-Rv2549c). In contrast, the related VapBC complexes Rv0065A-Rv0065 and Rv0616A-Rv0617 expressed in a soluble form and could be purified. Since Rv0065 is closely related to Rv0549c, sharing 36% amino acid identity and 50% amino acid similarity with Rv0549c (Fig. S2) and Rv0617 shares 38% sequence identity and 55% sequence similarity with Rv3320c, we reasoned that these VapCs could serve as useful models for analyzing the biochemical function of a toxic mycobacterial VapCs. Limited proteolysis of the VapBC complex resulted in selective degradation of the unstable VapB allowing purification of Rv0065 and Rv0617 alone. As shown in Fig. 8, these purified proteins show Mg^2+^-dependent sequence-selective RNase activity on a single-stranded RNA substrate. This RNase activity is completely inhibited when the VapC proteins are in complex with their cognate VapBs.

## Discussion

In this study, the function of a representative subset of mycobacterial VapBC modules was investigated in *M. smegmatis* and Mtb. VapC toxicity was initially assessed using a modular system in which *vapC* expression was either constitutive (in the absence of TetR) or conditionally regulated by TetR as a function of inducer concentration or the level of *tetR* expression. Of the VapCs tested, five were growth inhibitory in *M. smegmatis*, with Rv0549c being noticeably less toxic than Rv0595c, Rv2549c and Rv2829c when conditionally expressed, and Rv3320c only displaying toxicity when constitutively expressed. The five VapCs that were growth inhibitory in *M. smegmatis* demonstrated the same differential toxicity in wild type Mtb, with Rv0549c displaying only modest growth inhibition when expressed conditionally or constitutively, and Rv3320c being most toxic, but only when expressed constitutively.

Conditional expression of the three Mtb VapCs that were most toxic in *M. smegmatis* (Rv0595c, Rv2549c and Rv2829c) resulted in a rapid initial decline in bacterial CFUs, suggestive of VapC-mediated cell death or protracted bacteriostasis. Selection against VapC toxicity in *M. smegmatis* was revealed by loss or rearrangement of the expression vector to eliminate *vapC* expression in cells that survived toxic VapC exposure. Although the same three VapCs were growth inhibitory in Mtb, their effects were bacteriostatic in this host. However, a bactericidal effect in Mtb was observed by conditional expression of Rv2549c in a mutant strain that lacks the cognate Rv2550c antitoxin, confirming that VapC toxicity in Mtb can be tempered by expression if its cognate antitoxin from the corresponding *vapBC* module on the chromosome. In line with this observation, co-expression of the cognate *vapB* on an operon with *vapC* abrogated toxicity of all VapCs that were growth inhibitory in *M. smegmatis*. The specificity of toxin neutralization by the cognate VapB was demonstrated using an uncoupled system in which *vapC* and *vapB* genes were conditionally expressed under control of different regulatable promoters and from distinct chromosomal loci. In this assay, VapC-induced growth inhibition was specifically neutralized in *M. smegmatis* expressing the cognate *vapB*; in contrast, expression of non-cognate *vapBs* was ineffective. Together with the enhanced toxicity of Rv2549c in a mutant of Mtb that lacks the cognate antitoxin (Rv2550c), and the specificity of interaction between cognate VapB-VapC pairs revealed by Y2H analysis, our results argue against (functionally relevant) interactions between the toxin and antitoxin components of different VapBC modules in Mtb, consistent with findings from another study [34].

The lack of toxicity of Rv1953 under all conditions tested was consistent with the predicted abrogation of nuclease activity in this C-terminally truncated protein. In contrast, the reason for the lack of toxicity of Rv0627, Rv2010, Rv2546 and Rv2548 following expression in both Mtb and *M. smegmatis* was less clear. During the course of our study, the effects of toxin expression on the growth of *E. coli*, *M. smegmatis* and/or Mtb hosts were reported by other groups [38], [40], [43], [44]. Although some concordance exists, there are many differences between studies, even when the same host organism was employed for assessing toxicity. For example, Rv0627 was non-toxic in *M. smegmatis* and Mtb in our study, and in *M. smegmatis* in that of Ramage *et al*. [34], but this VapC was reported by Miallau *et al*. [31] as toxic in Mtb and *M. smegmatis*. Similarly, we found Rv0595c, Rv2549c and Rv3320c to be toxic in *M. smegmatis*, but Ramage *et al*. [34] did not, whereas the converse was true for Rv2010 and Rv2548 which were scored as toxic in that study, but not in the present one. Moreover, while Robson *et al*. reported growth inhibition of *M. smegmatis* by conditional expression of MSMEG_1284 [59], no such effect was observed in either wild type *M. smegmatis* or a deletion mutant lacking the *MSMEG_1283-MSMEG_1284* module in this study. These discrepancies may be due to differences in expression vectors, translation initiation signals, growth conditions and/or host strains used for VapC over-expression, which could result in assay-dependent biases. One or more of these factors may preclude the detection of toxin-induced growth inhibition if insufficient levels of the protein are produced following over-expression, leading to the incorrect classification of a subset of VapC as ‘non-toxic’. Our observations suggest that the reduced level of Rv2546 relative to Rv2549c might explain why this was non-toxic in our assay system, a notion substantiated by the finding that the toxicity of Rv2549c was only evident once the level of induced protein exceeded a certain threshold. Such factors clearly complicate distinguishing ‘functional’ TA modules from others, and suggest that the list of 30 modules defined as ‘toxic’ is likely an under-representation, biased by assay-dependent effects, as postulated [34].

The five VapCs identified as toxic in this study proved to be refractory to expression in a soluble form when complexed with their cognate VapCs, and as such, could not be isolated in a form suitable for biochemical analysis. Nonetheless, we were able to isolate and purify the Rv0549c homolog, Rv0065 and the Rv3320c homolog, Rv0617, respectively, and demonstrate that they have sequence-selective RNase activity. This finding strongly implies an association between VapC toxicity and translational inhibition as a result of RNA cleavage in mycobacteria, and moreover, suggests that the *vapBC* family provides a potentially abundant source of RNase activity in Mtb that might vary as a function of regulated expression of individual modules, and/or the rates of antitoxin degradation. The effects of extensive and variable nuclease activity on the biology of Mtb are likely to be profound and underscore the importance of identifying the cellular target(s) of VapCs in Mtb. Such studies are likely to provide valuable insights into the physiological role(s) that this family of proteins plays in the biology of this major human pathogen.

## Materials and Methods

### Bacterial strains, plasmids and culture conditions

The bacterial strains and plasmids used in this study are described in Table S4. Unless otherwise indicated, *M. smegmatis* strains were grown in Difco Middlebrook 7H9 media (BD) supplemented with 0.085 % NaCl, 0.2 % glucose, 0.2 % glycerol and 0.05 % Tween 80 or on solid Difco Middlebrook 7H10 media (BD) supplemented with 0.085 % NaCl, 0.2 % glucose and 0.5 % glycerol. Mtb strains were grown in Middlebrook 7H9 media supplemented with 0.2 % glycerol, Middlebrook oleic acid-albumin-dextrose-catalase (OADC) enrichment (Merck) and 0.05 % Tween 80. Hygromycin (Hyg), kanamycin (Km) and gentamycin (Gm) were used in mycobacterial cultures at final concentrations of 50, 25 and 5 µg/ml, respectively. ATc (Sigma) inducer was used at concentrations up to 200 ng/ml.

### Construction of deletion mutant strains

A 6097 bp *Eco*RI/*Asp*718 fragment carrying *Rv2009-Rv2010* was excised from BacD8 [64] and inserted in pGEM3Zf(+). An upstream homologous fragment was obtained by subcloning a 2455 bp *Pst*I fragment in pGEM3Z(+)f. A 3143 bp downstream homologous fragment was excised from the BacD8 subclone by digestion with *BsD*RI and *Pst*I and cloned in the *Xba*I site of the vector carrying the upstream fragment. The resulting deletion allele was excised as a 5654 bp *Hin*dIII/*Asp*718 fragment and inserted in p2NIL before introducing the *lacZ*-*sacB*-*hyg* cassette from pGOAL19 to produce p2Δ2009_10KO. This vector was used to construct a mutant of Mtb in which the last 78 amino acids of the antitoxin (Rv2009, containing the DNA binding motif) and all except the C-terminal 33 amino acids of the toxin (Rv2010) were removed. Suicide plasmids for generating other deletion mutants were constructed by PCR amplification from genomic DNA of upstream and downstream homologous sequences including the 5′ and 3′ termini of the gene of interest using primer pairs described in Table S5. Amplicons were cloned in pGEM3Z(+)f and sequenced before sub-cloning the corresponding upstream and downstream fragments in p2NIL to create deletion alleles. The *lacZ-sacB-hyg* cassette from pGOAL19 was inserted in the p2NIL subclones to create p2Δ0595cKO, and p2Δ2545_50cKO as suicide substrates for introducing unmarked deletions in Mtb *Rv0595c* and *Rv2545-Rv2550c*, and p2ΔSM1283_84KO as a substrate for generating an unmarked deletion in *MSMEG_1283-MSMEG_1284* (Table S4). Suicide vectors were electroporated into Mtb H37Rv or *M. smegmatis* mc^2^155 and allelic exchange mutants recovered by two-step selection, as described [65]. Mutant genotypes were confirmed by Southern blot analysis.

### Construction of vectors for conditional expression of toxins and antitoxins in mycobacteria

VapC toxin-encoding sequences were amplified by PCR from Mtb H37Rv chromosomal DNA using the primers listed in Table S6. In all cases, ribosome binding sites were changed to a standardized consensus sequence (GGAAG/A) in order to optimize the yield of expressed protein [66]. For cloning downstream of the P_myc1_ *tetO* promoter-operator element and ATc-dependent regulation of expression [60], Rv0549c, Rv0595c, Rv2829c, Rv1953 were expressed as *Bam*HI/*Pst*I fragments, Rv2546, Rv2548, Rv2549c were expressed as *Bam*HI/*Hind*III fragments, and Rv0627 was expressed as a *Pst*I-*Hind*III fragment and cloned in pSE100. The *MSMEG_1284* ORF was cloned in the *Pvu*II site of pSE100. Derivatives of Rv2546 and Rv2549c containing a C-terminal 3×FLAG epitope tag were expressed using the same forward primer as for the native version, and a modified reverse primer containing the 3× FLAG peptide-encoding sequence (Table S6). To ensure efficient translation of the expressed VapC proteins following ATc-induction, a consensus RBS (GGAAG/A) [66] was included upstream of the start codon of the *vapC* genes in those instances where an endogenous ribosome-binding site (RBS) was not clearly discernible. Other than this modification, the promoter architecture and start codons of the native *vapC* genes were preserved.

Vectors for expression of the *vapBC* modules *Rv0550c-Rv2549c*, *Rv0596c-Rv0595c*, *Rv2550c-Rv2549c* and *Rv2830c-Rv2829c* were generated using a *vapB*-specific forward primer and the reverse primer used for expression of the corresponding *vapC* (Table S6). All expression constructs were confirmed by DNA sequencing, and electroporated into *M. smegmatis* or Mtb alone or with an integrative *tetR*-containing plasmid in which *tetR* is expressed under control a strong (pMC1s) or intermediate strength promoter (pMC2m) [60]. Transformants were selected on 7H10 agar supplemented with Hyg or Hyg and Km respectively.

Vectors for uncoupled, regulated expression of the Rv2549c and either cognate (Rv2550c), or non-cognate VapB antitoxins (Rv2830c, Rv0596c) were constructed as follows. The L5-based integrative vector, pMC1s [60], was digested with *Not*I and the vector backbone re-ligated to generate pMC1r. The acetamide-inducible acetamidase promoter from *M. smegmatis* [62] was cloned in pMC1r to produce pMAP. The antitoxin-encoding genes, *Rv2550c*, *Rv0595c* and *Rv2830c*, were PCR-amplified (Table S6) and cloned in pMAP to produce pMAP2550c, pMAP0595c and pMAP2830c, respectively. The Tweety-based integration vector [61], pTTP1BG, was prepared by digesting pTTP1B [61] with *Hind*III, re-ligation of the vector and insertion of a Gm^R^ cassette [67] in the *Pst*I site. A *Not*I fragment from pMC1s carrying the P_smyc_-*tetR* element was cloned in the *Sma*I site of pTTP1BG followed by cloning of the 883-bp *Spe*I/*Cla*I fragment from pSE2549c which carries the P_myc1_ *tetO*::*Rv2549c* element in the *Eco*RI site of the resulting vector to produce pTT2549c. This vector was electroporated into *M. smegmatis* alone or together with each of the antitoxin-expressing pMAP subclones.

### Effect of conditional overexpression of VapCs on mycobacterial growth and viability

VapC toxicity was assessed both on solid medium and in liquid culture in *M. smegmatis* and in liquid culture only in Mtb. For the *M. smegmatis* plating assay, transformants were grown in Middlebrook 7H9 media containing Hyg and Km to an OD_600_ of approximately 1.0. The cultures were diluted in a 10-fold series and spotted on 7H10 plates with or without varying concentrations of ATc. The plates were incubated at 37°C and the growth checked after 24 and 48 h. For toxicity assessment in liquid culture, *M. smegmatis* transformants were grown in Middlebrook 7H9 media containing Km and Hyg to an OD_600_ of 0.1 - 0.4 and diluted into fresh warm media to an OD_600_ of 0.1. The cultures were split and either treated with ATc (25 ng/ml; induced) or left untreated (uninduced). The OD_600_ of each culture was measured every 2 h and viability scored by plating, in duplicate, serial dilutions of samples taken every 4 h over a period of 25 h.

Mtb transformants were grown in Middlebrook 7H9 media containing Hyg and Km to an OD_600_ of 0.1. The cultures were diluted to an OD_600_ of 0.04 and left to grow overnight before being split into two equal aliquots which were treated with ATc (25 or 50 ng/ml – induced) or left untreated (uninduced). Growth and viability were assessed by periodically monitoring OD_600_ and CFUs over 6 d.

### Stress sensitivity testing

The susceptibility of the *M. smegmatis vapBC* mutant to various stresses was tested using previously described methods [52], [68], [69], [70].

### Analysis of gene expression by RT-PCR

RNA was extracted from early log-phase cultures, as described [71]. Samples were treated twice with Turbo DNase (Ambion) and RT-PCR performed with the Phusion RT-PCR kit (Finnzymes) according to the manufacturer's instructions. Primers for RT-PCR analysis of expression of Mtb vapCs were designed using Primer 3 software (http://frodo.wi.mit.edu/cgi-bin/primer3/primer3_www.cgi) and are described in Table S6. Expression of *M. smegmatis sigA* was detected as described previously [72].

### Detection of FLAG-tagged proteins by Western blot analysis

*M. smegmatis* cells containing 3×FLAG-tagged VapC fusion proteins were grown in 90 ml cultures to mid log-phase (OD600∼0.4–0.5) and split equally. One 45-ml aliquot was treated with ATc (50 ng/ml) and the other served as the uninduced control. After 3 h induction, 20 ml of cells were harvested and resuspended in 250 µl of Bacterial Protein Extraction Reagent (B-PER II Reagent, Thermo Scientific) containing complete mini protease inhibitor cocktail (Roche). The cells were lysed three times for 20 s at speed 6 using the Savant Fastprep FP120 (BIO101), with 5 min intervals between pulses when the cells were cooled on ice. The protein concentration was determined using a Bradford assay and equivalent amounts of soluble and insoluble fractions of each induced and uninduced sample were resolved on an SDS-PAGE gel and the proteins transferred to a PVDF membrane (Amersham). The membrane was incubated with the HRP-conjugated mouse Anti-FLAG M2 antibody (Sigma) and the FLAG-tagged proteins proteins were detected using the ProteoQwest™ chemiluminescent Western blotting kit (Sigma).

### Y2H analysis

Y2H analysis was performed using the Clontech Matchmaker Y2H system and vectors carrying the VapCs, Rv0595c and Rv2549c, or VapBs Rv0596c, Rv2550c, and Rv2830c, cloned as *GAL-4* Activation Domain (AD) and/or Binding Domain (BD) fusions (Table S4). Interactions between selected VapC toxins and cognate vs. non-cognate VapB antitoxins were assessed according to the manufacturer's instructions.

### Expression, purification and biochemical analysis of recombinant Rv0065 and Rv00617

The ORFs encoding the *vapBC* operons Rv0065A-Rv0065 and Rv0616A-Rv0617 were amplified from Mtb H37Ra genomic DNA. Importantly, there are no differences in sequence between H37Rv and H37Ra in these regions. Moreover, the antitoxins for Rv0065 and Rv0617, which are designated herein as Rv0065A and Rv0617A, are not annotated in the H37Rv genome. However, these antitoxins are annotated in the H37Ra, BCG and CDC5115 genomes: Rv0065A corresponds to BCG0095A and Rv0617A corresponds to MT0645.2. The amplicons were digested with *Nco*I/*Hin*dIII restriction enzymes, purified and inserted into the pYUB28b shuttle vector [63] enabling expression of a C-terminal His-tag on VapC. The pYUBRv0065A-5 and pYUBRv0616A-6 constructs were then transformed into *M. smegmatis* mc^2^4517 cells. Rv0065A-Rv0065 and Rv0616A-Rv0617 VapBC complexes were expressed and purified as previously described for the VapBC complex from *M. smegmatis* [59]. VapBC complexes were digested with trypsin to remove VapB. Digestion reactions were stopped by addition of trypsin inhibitor from soybean and VapC was subsequently purified using anion exchange chromatography. RNA was transcribed from a ‘pentaprobe’ PCR product [73] using the T7 MEGAscript® kit (Ambion, USA) according to manufacturer's instructions. RNase activity assays for VapC proteins contained 1 µg purified VapC protein, 1 µg purified RNA, 12 mM sodium phosphate buffer, 6 mM NaCl and 6 mM MgCl_2_. Negative controls included addition of 12 mM EDTA and substitution of VapBC for VapC. Time course assay reactions were stopped by the addition of 10 µl formamide loading dye and heated to 70°C before loading onto a 10% urea-denaturing PAGE gel.

## Supporting Information

Figure S1 **Multiple sequence alignment of mycobacterial VapCs.** Sequences were aligned using the ClustalW2 multiple sequence alignment tool at the European Bioinformatics Institute website, http://www.ebi.ac.uk/Tools/clustalw2/index.html.
(PDF)

Figure S2 **Phylogenetic tree of VapC proteins from mycobacteria.** VapCs were aligned using ClustalW2 multiple sequence and server alignment server (http://www.ebi.ac.uk/Tools/clustalw2/index.html). The tree was generated in Jalview 2.08.1 based on percentage identity between sequences.
(PDF)

Table S1 Properties of mycobacterial *vapBC* toxin-antitoxin modules selected for study.
(PDF)

Table S2 VapC toxicity in *M. smegmatis* mc^2^155 assessed by transformation efficiency of VapC expression vector.
(PDF)

Table S3 VapC toxicity in Mtb assessed by transformation efficiency of VapC expression vector.
(PDF)

Table S4 Strains and plasmids used in this study.
(PDF)

Table S5 Oligonucleotides used to create knockout vectors for allelic exchange mutagenesis.
(PDF)

Table S6 Oligonucleotides used for expression vector construction, yeast two-hybrid (Y2H) vector construction and RT-PCR.
(PDF)
