# Supplementary material for: VapC Toxins from Mycobacterium tuberculosis Are Ribonucleases that Differentially Inhibit Growth and Are Neutralized by Cognate VapB Antitoxins
Source: PLoS One. 2011 Jun 29;6(6):e21738. doi: 10.1371/journal.pone.0021738 (PMC3126847; doi:10.1371/journal.pone.0021738)
Supplement: Figure S1 — Multiple sequence alignment of mycobacterial VapCs. Sequences were aligned using the ClustalW2 multiple sequence alignment tool at the European Bioinformatics Institute website, http://www.ebi.ac.uk/Tools/clustalw2/index.html. (PDF) [file pone.0021738.s001.pdf]

```
Conservation:                                                       8 56
Rv0624            1  -----------------------------------------VIDTSA------------LVAMLSDEPDA   17
MSMEG_1284        1  ----------------------------------------MVIDTSA------------LVAILTDEPDA   18
Rv2759c           1  ----------------------------------------VIVDTSA------------IVAIVSGESGA   18
Rv0609            1  ----------------------------------------VIVDTSA------------IIAILRDEDDA   18
Rv1982c           1  ----------------------------------------MIVDTSA------------VVALVQGERPH   18
Rv0582            1  ----------------------------------------VIIDTSA------------LLAYFDAAEPD   18
Rv1561            1  ---------------------------------------MILIDTSA------------WVEYFRATGSI   19
Rv0065            1  -------------------------------------VDECVVDAAA------------VVDALAGKGAS   21
Rv3408            1  ---------------------------------------VIYMDTSA------------LTKLLISEPET   19
Rv3384c           1  -------------------------------------MAAIYLDSSA------------IVKLAVREPES   21
Rv1962c           1  ---------------------------------------VIYLETSA------------LVKLIRIEVES   19
Rv2231            1  VLWILGPHTGPLLFDAVASLDTSPLAAARYHGDQDVAPGVLDFAVNVRHDRPPEWLVRQLAALLPELARY   70
Rv0665            1  --------------------------------VTEGEVGVGLLDTSV------------FIARESGGAI-   25
Rv0627            1  ---------------------------------VSTTPAAGVLDTSV------------FIATESGRQLD   25
Rv0595c           1  -----------------------------------VNVRRALADTSV------------FIGIEATRFDP   23
Rv1720c           1  ---------------------------------------VIVLDASA------------AVELMLTTPAG   19
Rv0960            1  ---------------------------------------MIVVDASA------------ALAALLND---   16
Rv0661c           1  ---------------------------------------MIVLDTTV------------LVYAKGAEHPL   19
Rv0549c           1  -----------------------------VRASPTSPPEQVVVDASA------------MVDLLARTSDR   29
Rv0656c           1  ------------------------------------------------------------LAAATTTGTH   10
Rv2596            1  ---------------------------------------VIAPDTSV------------LVAGFATWHEG   19
Rv0598c           1  ------------------------------------VKPPLAVDTSV------------AIPLLVRTHTA   22
Rv2548            1  ---------------------------------------VKLIDTTI------------AVDHLRGEPA-   18
Rv2103c           1  ---------------------------------------MKIVDANV------------LLYAVNTTSEH   19
Rv1397c           1  ---------------------------------------MILVDSDV------------LIAHLRGVV--   17
Rv0617            1  --------------------------------------VTVLLDANV------------LIALVVAEHVH   20
Rv1838c           1  ---------------------------------------VILVDSNI------------PMYLVGASHPH   19
Rv2010            1  ----------------------------------------MIVDTSV------------WIAYLSTSES-   17
Rv1114            1  ---------------------------------------VILVDTSV------------WIEHLRAADA-   18
Rv2602            1  ---------------------------------------MLLCDTNI------------WLALALSGHVH   19
Rv3180c           1  ---------------------MTPNAASTGDSAKNTITGCCLITARA------------LVARTRSISLP   37
Rv3697c           1  --------------------------------------VSETFDVDV------------LVHATHRASPF   20
Rv3320c           1  --------------------------------------MRALLDVNV------------LLALLDRDHVD   20
Rv2530c           1  --------------------------------------VTALLDVNV------------LIALGWPNHVH   20
Rv2494            1  ---------------------------------------VALLDVNA------------LVALAWDSHIH   19
Rv2872            1  ---------------------------------------MLCVDVNV------------LVYAHRADLRE   19
Rv0749            1  ---------------------------------------MFLLDANV------------LLAAHRGDHPN   19
Rv0277c           1  ---------------------------------------MFLIDVNV------------LLAAHRGDHPN   19
Rv2829c           1  -------------------------------------MTTVLLDSHV------------AYWWSAEPQRL   21
Rv1242            1  ---------------------------------------VIIPDINL------------LLYAVITGFPQ   19
Rv2863            1  ---------------------------------------MIFVDTNV------------FMYAVGRDHPL   19
Rv2549c           1  ---------------------------------------MIFVDTSF------------WAALGNAGDAR   19
Rv1953            1  --------------------------------------VTYVLDTNV------------VSALRVPGRH-   19
Rv2757c           1  ------------------------------------MTTRYLLDKSA------------AYRAHLPAV--   20
Rv2546            1  --------------------------------------MVFCVDTSA------------WHHAARPEV--   18
Rv0301            1  -----------------------------------VTDQRWLIDKSA------------LVRLTDSPD--   21
Rv2527            1  -------------------------------------MTTWILDKSA------------HVRLVAGAT--   19
Rv0240            1  ---------------------------------------VLSIDTNI------------LLYAQNRDCPE   19
Consensus aa:        ........................................hhlDhsh............hh.h.......
Consensus ss:                                                eeeehhh            hhhhh

Conservation:                                                             6
Rv0624           18  -------ERFEAAVEA--------------------------DHIRLMSTASYLETALVIEARF------   48
MSMEG_1284       19  -------ELLEGAVAD--------------------------DPVRTMSTASYLETAIVIESRF------   49
Rv2759c          19  -------QVLKEALER--------------------------SPNSRMSAPNYVELCAIMQRRD------   49
Rv0609           19  -------AAYADALAN--------------------------ADVRRLSAASYLECGIVLDSQR------   49
Rv1982c          19  A------TLVAAALAG--------------------------AHSPVMSAPTVAECLIVLTARH------   50
Rv0582           19  H------AAVSECIDSS-------------------------ADALVVSPYVVAELDYLVATRV------   51
Rv1561           20  A------AVEVRRLLSEE------------------------AARIAMCEPIAMEILSGALD--------   51
Rv0065           22  A------IVLRGLLKE---------------------------SISNAPHLLDAEVGHALRRAVLSDEI-   57
Rv3408           20  -------TELRTWLTAQSG----------------------QGEDAATSTLGRVESMRVVARYGQP----   56
Rv3384c          22  -------DALRRYLRT--------------------------RHPRVSSALARAEVMRALLDKGE-----   53
Rv1962c          20  -------DALADWLDDR------------------------TELRWITSALTEVELSRAIRAVSP-----   53
Rv2231           71  PST-DDVHRAQDAVAERHGRTRDEVLPLVGAAEGFALLHNLSPVRAAIVVPAFTEPAIALSAAGITAHHV  139
Rv0665           26  -------------ADL--------------------------PERVALSVMTIGELQLGLLNAGDS----   52
Rv0627           26  ------------EALI--------------------------PDRVATTVVTLAELRVGVLAAATT----   53
Rv0595c          24  ------------DRFA--------------------------GYEWGVSVVTLGELRLGVLQASGP----   51
Rv1720c          20  -------AAVAR-RLR--------------------------GETVHAPAHFDVEVIGAIRQAVVRQL--   53
Rv0960           17  -------GQARQ-LIA--------------------------AERLHVPHLVDSEIASGLRRLAQRDR--   50
Rv0661c          20  R------DPCRDLVAAIAD----------------------ERIAATTTAEVIQEFVHVRARRR------   55
Rv0549c          30  C------SAVRA-RLA--------------------------RTAMHAPAHFDAEVLSALGRMQRAGA--   64
Rv0656c          11  -------RGLEL-RAA--------------------------QRAVGSCEPQRAEFCRSARN--------   38
Rv2596           20  H------EAAVRALNR----------------------------GVHLIAHAAVETYSVLTRLPPPHRI-   54
Rv0598c          23  H------AAVVAWWAH---------------------------REAALCGHALAETYSVLTRLPRDLRL-   58
Rv2548           19  ---------AAVLLAELIN----------------------NGEEIAASELVRFELLAGVRES-------   50
Rv2103c          20  H------KPSLRWLDGALS----------------------GADRVGFAWVPLLAFVRLATKVGLFPRPL   61
Rv1397c          18  --------AARDWLVSAR-----------------------KDGPLAISVVSTAELIGGMR---------   47
Rv0617           21  H------DAAADWLMAS-------------------------DTGFATCPMTQGSLVRFLVRSGQ-----   54
Rv1838c          20  K------LDAQRLLESALS----------------------GGERLVTDAEVLQEICHRYVAI-------   54
```

```
Rv2010            18  --------LASRWLADRIA----------------------ADSTVIVPEVVMMELLIGKTDE-------  50
Rv1114            19  --------RLVELL---------------------------GDDEAGCHPLVIEELALGSIKQ-------  46
Rv2602            20  H------RASRAWLDTIN-----------------------APGVIHFCRATQQSLLRLLTNRTVLGAYG  60
Rv3180c           38  GMPFRMPADYHNASSD--------------------------EPTNRHPWPAPARCCRHE----------  71
Rv3697c           21  H------DKAKTLVERFLA----------------------GPGLVYLLWPVALGYLRVVTHPTLLGAPL  62
Rv3320c           21  H------ERARAWITGQI------------------------ERGWASCAITQNGFVRVISQPRYPSPI-  59
Rv2530c           21  H------AAAQRWFTQFS------------------------SNGWATTPITEAGYVRISSNRSVMQVST  60
Rv2494            20  H------ARIREWFTANA------------------------TLGWATCPLTEAGFVRVSTNPKVLPSAI  59
Rv2872            20  H------ADYRGLLERLAN----------------------DDEPLGLPDSVLAGFIRVVTNRRVFTEPT  61
Rv0749            20  H------RTVRPWFDRLLA----------------------ADDPFTVPNLVWASFLRLATNRRIFEIPS  61
Rv0277c           20  H------RTVRPWFDRLLA----------------------ADDPFTVPNLVWASFLRLTTNRRIFEIPS  61
Rv2829c           22  S------MAASQAIEH--------------------------ADELAVAAISWFELAWLAEQERIQL---  56
Rv1242            20  H------RRAHAWWQDTVN----------------------GHTRIGLTYPALFGFLRIATSARVLAAPL  61
Rv2863            20  R------MPAREFLEHSLE----------------------HQDRLVTSAEAMQELLNAYVPVGR-----  56
Rv2549c           20  H------GTAKRLWASK-------------------------PPVVMTSNHVLGETWTLLNRRCG-----  53
Rv1953            20  -------PAVAAWADSV------------------------QVAEQFVVAITLAEIERGVIAKER-----  53
Rv2757c           21  ---------RHRLEPLM------------------------ERGLLARCGITDLEFGVSARSR-------  50
Rv2546            19  ---------ARRWLAAL------------------------SADQIGICDHVRLEILYSANSA-------  48
Rv0301            22  ---------MEIWSNRI------------------------ERGLVHITGVTRLEVGFSAECGE------  52
Rv2527            20  -------------PPAGI-----------------------DLTDLAICDIGELEWLYSARSA-------  46
Rv0240            20  H------DAAAAFLVECA-----------------------GRADVAVCELVLMELYQLLRNPTVVTRPL  60
Consensus_aa:         .........h..hh...............................h.hs..h..ph...h..........
Consensus_ss:                hhhhhhhhh                             eee hhhhhhhhhhhh


Conservation:
Rv0624            49  ------------------------------GEPGGRELDLWL--------HRA--------AVDLVAVHA  72
MSMEG_1284        50  ------------------------------GEPGGRELDLWL--------HRA--------SVALVAVDA  73
Rv2759c           50  ------------------------------RPEISRLVDRLL--------DDY--------GIQVEAVDA  73
Rv0609            50  ------------------------------DPVISRALDELI--------EEA--------EFVVEPVTE  73
Rv1982c           51  ------------------------------GPVARTIFERLR--------SEI--------GLSVSSFTA  74
Rv0582            52  ------------------------------GVDAELAVLREL--------AGG--------AWELANCGA  75
Rv1561            52  -------------------------------DNTHTTLERLV--------NGL--------PSLNV-DDA  73
Rv0065            58  ------------------------------SEEQARAALDAL--------PYL--------IDNRYPHSP  81
Rv3408            57  --------------------------------GQTERARYLL--------DGL----------DILPLTE  76
Rv3384c           54  --------------------------------SARKAGRRAL--------AHL----------DLLRVDK  73
Rv1962c           54  --------------------------------EGLPAVPSVL--------ARL----------DRFEIDA  73
Rv2231           140  VLKPPFVLDTAHVPDDADLVVVGNPTNPTSVLHLREQLLELR--------RPG----------RILVVDE  191
Rv0665            53  -----------------------------ATRSRRADTLALA--------RTA----------DQIPVSE  75
Rv0627            54  -----------------------------DIRAQRLATLESV--------ADM----------ETLPVDD  76
Rv0595c           52  -----------------------------EAAARRLSTYQLA--------QRF----------EPLGIDE  74
Rv1720c           54  -----------------------------ISDHEGLVVVVNF--------LSL--------PVRRWPLK-  77
Rv0960            51  -----------------------------LGAADGRRALQTW--------RRL--------AVTRYPVV-  74
Rv0661c           56  ------------------------------DRSDAA-ALGRVTMPNCSRRYSPSIEATSKRGLTLFETTP  94
Rv0549c           65  -----------------------------LTVAYVDAALEEL--------RQV--------PVTRHGLSS  89
Rv0656c           39  -------------------------------ADEFDQMSRMF--------GDV---------YPDVPVPK  60
Rv2596            55  -----------------------------APVAVHAYLADIT-------------------SSNYLALDA  76
Rv0598c           59  -----------------------------APMDAARLLTERF--------------------AAPLLLSS  79
Rv2548            51  ---------------------------------ELAALEAFF--------SAV----------VWTLVTE  69
Rv2103c           62  -----------------------------PREAAITQVADWL--------AAP--------SAVLVNPTV  86
Rv1397c           48  -------------------------------TAERREVWRLL--------ASF----------RVQPATE  68
Rv0617            55  -----------------------------SAAAARDVVSAVQ--------CTS--------RHEFWPDAL  79
Rv1838c           55  -----------------------------KRREAIQPAFDAI--------IGV--------VDEVLPIER  79
Rv2010            51  --------------------------------DTAALRRRLL--------QRF----------AIEPLAP  70
Rv1114            47  ----------------------------------RDVVLDLL--------ANL----------YQFPVVT  64
Rv2602            61  SP-------------------------PLTNREAWAAYAAFL--------DDD--------RIVLAGAEP  89
Rv3180c           72  -------------------------------------WRTMRRTN--ACDRRR--------FGLSLTIHE  94
Rv3697c           63  -----------------------------APEVAVENIEQFT--------SRP--------HVRQVGEAN  87
Rv3320c           60  -----------------------------SVAHAIDLLARAT--------HTR--------YHEFWSCTV  84
Rv2530c           61  -----------------------------TPAIAIAQLAAMT--------SLA--------GHTFWPDDV  85
Rv2494            60  -----------------------------GIADARRVLVALR--------AVG--------GHRFLADDV  84
Rv2872            62  -----------------------------SPQDAWQAVDALL--------AAP--------AAMRLRPGE  86
Rv0749            62  -----------------------------PRAEAFAFVEAVT--------AQP--------HHLPTNPGP  86
Rv0277c           62  -----------------------------PRADAFAFVEAVN--------AQP--------HHLPTSPGP  86
Rv2829c           57  ------------------------------AIPVLSWLQQLA--------E----------HVRTVGITP  78
Rv1242            62  -----------------------------PTADAIAYVREWL--------SQP--------NVDLLTAGP  86
Rv2863            57  -------------------------------NSTLDSALTLV--------RA---------LTEIWPVEA  78
Rv2549c           54  -------------------------------HRAAVAAAAIR--------LST--------VVRVEHVTA  76
Rv1953            54  -----------------------------TDPTQSEHLRRWF-------------------DDKVLRIFV  75
Rv2757c           51  -------------------------------EDHRTLGTYRR--------DAL----------EYVNTPD  71
Rv2546            49  --------------------------------TDYDALADEL--------DGL----------ARIPVGA  68
Rv0301            53  -------------------------------IARREFREPPL--------SAM----------PVEYLTP  73
Rv2527            47  --------------------------------TDYDSQQTSL--------RAY----------QILRAPS  66
Rv0240            61  -----------------------------EGPEAAEVCQTFR--------RNR--------RWALLENAP  85
Consensus_aa:         .....................................h...........................h.hs.
Consensus_ss:                                        hhhhhhhhhhh        h            eee   h


Conservation:
Rv0624            73  ---------------------------------------------DQADAARAA---YRTYGKGRH--RA  92
MSMEG_1284        74  ---------------------------------------------DQADAARLA---YRRYGKGRH--RA  93
```

```
Rv2759c        74  ---------------------------------------------DQARVAAQA---YRDYGRGSG-HPA   94
Rv0609         74  ---------------------------------------------RQARLARAA---YADFGRGSG-HPA   94
Rv1982c        75  ---------------------------------------------EHAAATQRA---FLRYGKGRH--RA   94
Rv0582         76  ---------------------------------------------AEIEQAARI---VTKYQDQ------   91
Rv1561         74  ---------------------------------------------IDFRAAAGIYRAARRAGET------   92
Rv0065         82  ---------------------------------------------RLIEYTW-----QLRH---------   92
Rv3408         77  ---------------------------------------------PVIGLAETI---GP----------A   88
Rv3384c        74  ---------------------------------------------RVLDLAGGL---LP----------F   85
Rv1962c        74  ---------------------------------------------VIRSTAAAY---PN----------P   85
Rv2231        192  AFADWVPGEPQSLADDSLPDVLVLRSLTKTWSLAGLRVGYALGSPDVLARLTVQ--------RAH----W  249
Rv0665         76  ---------------------------------------------AVMISLARL---VADCRAAG----V   93
Rv0627         77  ---------------------------------------------DAARMWARL---RIHLAESG----R   94
Rv0595c        75  ---------------------------------------------AVSEAWALL---VSKLRAAK----L   92
Rv1720c        78  ---------------------------------------------PFTQRAYQL---R-----------S   88
Rv0960         75  ---------------------------------------------GLFERIWEI---R-----------A   85
Rv0661c            ----------------------------------------------------------------------
Rv0549c        90  ----------------------------------------------LLAGAWSR---RD-----------   99
Rv0656c        61  ---------------------------------------------SVWRWIDSA---QHRLARAGA--VG   80
Rv2596         77  ---------------------------------------------CSYRGLTDH---LAEHDVT------   92
Rv0598c        80  ---------------------------------------------RTTEHLPRV---LAQFEIT------   95
Rv2548         70  ---------------------------------------------DIARIGGRL---ARRYRSSH----R   87
Rv2103c        87  ---------------------------------------------RHADILARM---LTYVGTG------  102
Rv1397c        69  ---------------------------------------------VIARRAGDM---MRRYRRSH----N   86
Rv0617         80  ---------------------------------------------SFAGVEVAG---------VV----G   91
Rv1838c        80  ---------------------------------------------TDVEHARDA---LLRYQ--------   93
Rv2010         71  --------------------------------------------VRDAEDAAAI---HRRCRRGG----D   89
Rv1114         65  -----------------------------------------------HDEVLRL---VGRRRLWG----R   80
Rv2602         90  ---------------------------------------------DGLEAQWRA---FAVRQSP-----A  106
Rv3180c        95  ---------------------------------------------DACR---------------------   98
Rv3697c        88  ---------------------------------------------GFWPVYRRV---ADPVKPR------  103
Rv3320c        85  ---------------------------------------------SILD-SKVI---DRSRLHS------   99
Rv2530c        86  ---------------------------------------------PLIV-GSAG---DRDAVSN------  100
Rv2494         85  ---------------------------------------------SLVDDD-------VPLIVG------   96
Rv2872         87  ---------------------------------------------RHWMAFRQL---ASDVDAN------  102
Rv0749         87  ---------------------------------------------RHLMLLRKL---CDEADAS------  102
Rv0277c        87  ---------------------------------------------RHLVLLRKL---CDEADAS------  102
Rv2829c        79  ---------------------------------------------SVAATAVAL---PSSF---------   91
Rv1242         87  ---------------------------------------------RHLDIALGL---LDKLGTA------  102
Rv2863         79  ---------------------------------------------ADVAHARTL---HHRHP--------   92
Rv2549c        77  ---------------------------------------------DLEEQAWEW---LVRHDER------   92
Rv1953         76  ---------------------------------------------FARRGTNL-----------------   83
Rv2757c        72  ---------------------------------------------TVWVRAWEI---QEALTDKGF--HR   91
Rv2546         69  ---------------------------------------------ETFTRACQV---QRELAHVAGLHHR   90
Rv0301         74  ---------------------------------------------RIEDRALEV---QTLLADRGH--HR   93
Rv2527         67  ---------------------------------------------DIFDRVRHL---QRDLAHHRG-MWH   87
Rv0240         86  VMNEVW---------------------------------VLAATPRIARRR-------------------  103
Consensus_aa:      ...............................................h..h..h................
Consensus_ss:                                                   hhhhhhhhh   hhh

Conservation:                                      9       5                 6
Rv0624         93  GLN---------------------------YGDC-FSYGLAKISGQ-------PLLFKG----EDFQ-HT  122
MSMEG_1284     94  GLN---------------------------YGDC-FSYALAKVSGQ-------PLLFKG----EAFR-LT  123
Rv2759c        95  RLN---------------------------LGDT-YSYALAQVTGE-------PLLFRG----DDFT-HT  124
Rv0609         95  GLN---------------------------FGDC-LSYALAIDRRE-------PLLWKG----NDFG-HT  124
Rv1982c        95  ALN---------------------------FGDC-MTYATAQLGHQ-------PLLAVG----NDFP-QT  124
Rv0582         92  RIG---------------------------IADA-ANVVLADRYRTR------TILTLD----RRHFSAL  123
Rv1561         93  VRS---------------------------INDC-LIAALAIRHGA-------RIVHRD----ADFD-VI  122
Rv0065         93  NVT---------------------------FYDA-LYVALATALDV-------PLLTGD----SRLA-AA  122
Rv3408         89  TLR---------------------------SLDA-IHLAAAAQIKREL----TAFVTYD----HRLL-SG  121
Rv3384c        86  ELR---------------------------TLDA-IHLATAQRLGVDL----GRLCTYD----DRMR-DA  118
Rv1962c        86  ALR---------------------------SLDA-IHLATAQTAGSVAP--LTALVTYD----NRLK-EA  120
Rv2231        250  PLGTLQLTAIAACCAPRAVAAAAADAVRLTALRA-EMVAGLRSVGAEVVDGAAPFVLFNIADADGLR-NY  317
Rv0665         94  RRSV-------------------------KLTDA-LIAATAEIKV-------------------------  112
Rv0627         95  RVR---------------------------INDL-WIAAVAASRAL-------PVITQD----DDFA-AL  124
Rv0595c        93  RVP---------------------------INDS-WIAATAVAHGI-------AILTQD----NDYA-AM  122
Rv1720c        89  THT---------------------------VADG-AYVALAEGLGV-------PLITCD----GRLA-QS  118
Rv0960         86  NLS---------------------------AYDA-SYVALAEALNC-------ALVTAD----LRLS-DT  115
Rv0661c        95  GLE---------------------------ACDA-VLAAVAASAGAT------ALVSAD----PAFA-DL  125
Rv0549c       100  TLR---------------------------LTDA-LYVELAETAGL-------VLLTTD----ERLA-RA  129
Rv0656c        81  ALS---------------------------VVDL-LICDTAAARGL-------VVLHDD----ADYE-LA  110
Rv2596         93  GGA---------------------------TYDA-LVGFTAKAAGA-------KLLTRD----LRAV-ET  122
Rv0598c        96  GGA---------------------------VYDA-LVALAAAEHRA-------ELATRD----ARAK-DT  125
Rv2548         88  GID---------------------------DVDY-LIAATAIVVDA-------DLLTTN----VRHFPMF  118
Rv2103c       103  ANL---------------------------VNDA-HLAALAVEHRA-------SIVSYD----SDFG-RF  132
Rv1397c        87  RIG---------------------------LGDY-LIAATADVQDL-------QLATLN----VWHFPMF  117
Rv0617         92  HRQ---------------------------VTDA-YLAQLARSHDG-------QLATLD----SGLAHLH  122
Rv1838c        94  TLS---------------------------ARDA-LHIAVMAHHDIT------RLMSFD----RGFD-SY  124
Rv2010         90  TVR--------------------------SLIDC-QVAAMALRIGV-------AVAHRD----RDYE-AI  120
Rv1114         81  GLG---------------------------AVDANLLGSVALVGGA-------RLWTRD----KRLK-AA  111
Rv2602        107  PKV---------------------------WMDA-YLAAFALTGGF-------ELVTTD----TAFT-QY  136
```

```
Rv3180c          99  ------------------------------IISV-VPVVLEVRRAEP-----AHPATPY---PEPLA-RC  128
Rv3697c         104  GNL---------------------------VPDA-HLVALMRHHGIA------TIWSHD----RDFR-KF  134
Rv3320c         100  PKQ---------------------------VTDA-YLLALAVAHDG-------RFVTFD----QSIA-LT  129
Rv2530c         101  HRR---------------------------VTDC-HLIALAARYGG-------RLVTFD----AALA-DS  130
Rv2494           97  YRQ---------------------------VTDA-HLLTLARRRGV-------RLVTFD----AGVF-TL  126
Rv2872          103  GND---------------------------IADA-HLAAYALENNA-------TWLSAD----RGFA-RF  132
Rv0749          103  GDL---------------------------IPDA-VLAAIAVGHHC-------AVVSLD----RDFA-RF  132
Rv0277c         103  GDL---------------------------IPDA-VLGAIAVEHHC-------AVVSLD----RDFA-RF  132
Rv2829c          92  PGD---------------------------PADR-LIYATAIEHGW-------RLVTKD----RRLR-SH  121
Rv1242          103  SHL---------------------------TTDV-QLAAYGIEYDA-------EIHSSD----TDFA-RF  132
Rv2863           93  GLG---------------------------ARDL-LHLACCQRRGVT------RIKTFD----HTLA-SA  123
Rv2549c          93  EYS---------------------------FVDA-TSFAVMRKKGIQ------NAYAFD----GDFS--A  122
Rv1953           84  -------------------------------------IMQPLAGHI-------GYSLYS----GISW-F-  103
Rv2757c          92  SVK---------------------------IPDL-IIAAVAEHHGI-------PVMHYD----QDFE-RI  121
Rv2546           91  SVK---------------------------IADL-VIAAAAELSGT-------IVWHYD----ENYD-RV  120
Rv0301           94  GPS---------------------------IPDL-LIAATAELSGL-------TVLHVD----KDFD-AI  123
Rv2527           88  RTP---------------------------LPDL-FIAETALHHRA-------GVLHHD----RDYK-RI  117
Rv0240          104  ------------------------------LFDA-RLALTLRHHGVD------EFATRN----INGFTDF  132
Consensus aa:        ..............................h.Dh.hhhhht.............hhh.s......h...h
Consensus ss:                                      hhhh hhhhhhhh          eeeee      hhh hh


Conservation:
Rv0624          123  DIATVALP----------------------------------------------  130
MSMEG_1284      124  DVAAVH------------------------------------------------  129
Rv2759c         125  DIRPACT-----------------------------------------------  131
Rv0609          125  GVQRALDRR---------------------------------------------  133
Rv1982c         125  DLEFRGVVG------YWPGVA---------------------------------  139
Rv0582          124  RPIGGGRFT------VIP------------------------------------  135
Rv1561          123  ARIT-NLQA------ASFR-----------------------------------  134
Rv0065          123  PGLPCEIKL------VR-------------------------------------  133
Rv3408          122  CREV-GFVT------ASPGAVR--------------------------------  136
Rv3384c         119  AKTL-GMAV------IAPS-----------------------------------  130
Rv1962c         121  AEAL-SLAV------VAPGQAR--------------------------------  135
Rv2231          318  LQSK-GIAV------RRGDTFVGLDARYLRAAVRPEWPVLVAAIAEWAKRGGRR  364
Rv0665               ------------------------------------------------------
Rv0627          125  DG----AAS------VEIIRV---------------------------------  135
Rv0595c         123  PD----VEV------ITI------------------------------------  130
Rv1720c         119  HGHNAEIEL------VA-------------------------------------  129
Rv0960          116  GQAQCPITV-------VPR-----------------------------------  127
Rv0661c         126  SDVV-HVIPDA-AGMVSLLGDR--------------------------------  145
Rv0549c         130  WPSA-HAIG---------------------------------------------  137
Rv0656c         111  ERHL-PDIR-----VRRVVSADD-------------------------------  127
Rv2596          123  YERL-RVEV------ELVT-----------------------------------  134
Rv0598c         126  YEKI-GVHV------VVAA-----------------------------------  137
Rv2548          119  PDL------------QPPY-----------------------------------  125
Rv2103c         133  EGVRW----------DQPPALL--------------------------------  144
Rv1397c         118  EQLK--PPF------AVPGHRPRA------------------------------  133
Rv0617          123  GDVAV----------LIPTTT---------------------------------  133
Rv1838c         125  PGIKRLA-----------------------------------------------  131
Rv2010          121  RTHC-GLRT------EPLF-----------------------------------  132
Rv1114          112  CAES-GVAL------AEEVS----------------------------------  124
Rv2602          137  GGIE--LRL------LAK------------------------------------  146
Rv3180c         129  SRSP-GLNESSHMSGRIPP-----------------------------------  146
Rv3697c         135  EGIRI----------RDPFSG---------------------------------  145
Rv3320c         130  AVPGATKQH------LATL-----------------------------------  142
Rv2530c         131  ASAGL----------VEVL-----------------------------------  139
Rv2494          127  AQQRPKTPVE----LLTIL-----------------------------------  141
Rv2872          133  RRLRW----------RHPLDGQTHL-----------------------------  147
Rv0749          133  ASVRH----------IRPPL----------------------------------  142
Rv0277c         133  ASVRH----------IRPPI----------------------------------  142
Rv2829c         122  RHPRP----------VTVW-----------------------------------  130
Rv1242          133  ADLKW----------TDPLRE---------------------------------  143
Rv2863          124  FRS---------------------------------------------------  126
Rv2549c         123  AGFVE----------VRPE-----------------------------------  131
Rv1953               ------------------------------------------------------
Rv2757c         122  AAIT-RQPV------EWVVAPGTA------------------------------  138
Rv2546          121  AAIT-GQPT------EWIVPRGTL------------------------------  137
Rv0301          124  AALT-GQKT------ERLTHRPPSA-----------------------------  141
Rv2527          118  AVVRPGFQA-----CELSRGR---------------------------------  133
Rv0240          133  GFSR-VWD--------PITSDG--------------------------------  145
Consensus aa:        ......................................................
Consensus ss:
```

Fig. S1. Multiple sequence alignment of mycobacterial VapCs. Sequences were aligned using the ClustalW2 multiple sequence alignment tool at the European Bioinformatics Institute website, http://www.ebi.ac.uk/Tools/clustalw2/index.html
