# Supplementary material for: VapC Toxins from Mycobacterium tuberculosis Are Ribonucleases that Differentially Inhibit Growth and Are Neutralized by Cognate VapB Antitoxins
Source: PLoS One. 2011 Jun 29;6(6):e21738. doi: 10.1371/journal.pone.0021738 (PMC3126847; doi:10.1371/journal.pone.0021738)
Supplement: Figure S2 — Phylogenetic tree of VapC proteins from mycobacteria. VapCs were aligned using ClustalW2 multiple sequence and server alignment server (http://www.ebi.ac.uk/Tools/clustalw2/index.html). The tree was generated in Jalview 2.08.1 based on percentage identity between sequences. (PDF) [file pone.0021738.s002.pdf]

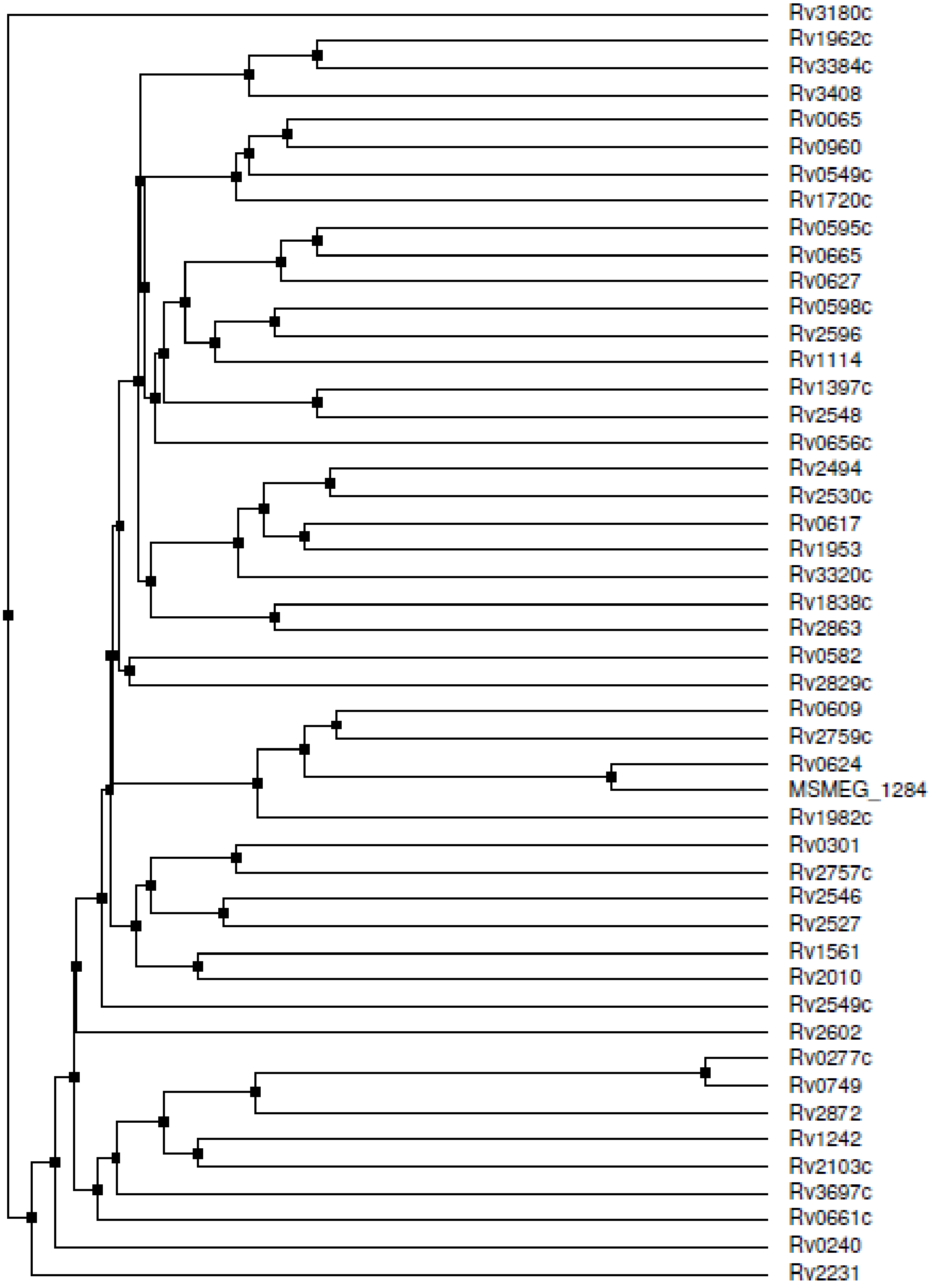

Fig. S2. Phylogenetic tree of VapC proteins from mycobacteria.
VapC proteins were aligned using ClustalW2 multiple sequence and server alignment server (http://www.ebi.ac.uk/Tools/clustalw2/index.html). The tree was generated in Jalview 2.08.1 based on percentage identity between sequences.
