# Supplementary material for: VapC Toxins from Mycobacterium tuberculosis Are Ribonucleases that Differentially Inhibit Growth and Are Neutralized by Cognate VapB Antitoxins
Source: PLoS One. 2011 Jun 29;6(6):e21738. doi: 10.1371/journal.pone.0021738 (PMC3126847; doi:10.1371/journal.pone.0021738)
Supplement: Table S1 — Properties of mycobacterial vapBC toxin-antitoxin modules selected for study. (PDF) [file pone.0021738.s003.pdf]

Table S1. Properties of mycobacterial *vapBC* toxin-antitoxin modules selected for study

Gene	In vitro essentiality[a]	Other properties and/or features
**Antitoxin (VapB)**		
Rv0550c	No [1]	Induced by heat stress [2], during macrophage infections [3] and high concentrations of vancomycin [4]
Rv0596c	-	Induced in a *sigE* mutant after SDS stress [5]
Rv0626	-	Induced *in vivo* but not *in vitro* [6]; repressed during infection of human macrophage-like cells [7]; and structure of C-terminal region determined in complex with toxin Rv0627 [8]
Rv1952	No [1]	Induced during phosphate starvation [9] and during macrophage infections [7]
Rv2009	No [1]	Induced during human macrophage infections [3,10], in SCID mice [6], after SDS stress [5] and during transition to hypoxia [3]; but repressed during nutrient starvation [11], hypoxia [12], and in wild type H37Rv vs. a *phoP* mutant [13]; Part of a genomic island [3]
Rv2545	No [1]	Repressed at low pH *in vitro* [14]; and a P19L polymorphism was identified in MDR strain of Mtb [15]
Rv2547	No [1]	Induced during hypoxia [3,16] and infection of macrophages [3,17]
Rv2550c	No [1,18]	Induced during macrophage infections [17], in Balb/c mice [6], in the presence of high iron concentrations [19] and SDS stress [5]; but repressed by hypoxia [20] and in a *sigE* mutant after SDS stress [5]
Rv2830c	Yes [1]	Induced during hypoxia [21], SDS stress [5] and during *in vitro* and *in vivo* growth [6]
Rv3321c	No [1]	Induced in SCID mice [6] and during human macrophage infections [10]
MSMEG_1283	-	-
**Toxin (VapC)**		
Rv0549c	No [1]	Induced by hypoxia [3,16], SDS stress [5], during adaptation to nutrient starvation [22], during infection of human macrophages [3,10], and in the presence of high concentrations of vancomycin [4]. Non-toxic in *E. coli* [23] but toxic in *M. smegmatis* [3]
Rv0595c	No [1]	Required for survival in nonhuman primate lungs [24]; induced during adaptation to nutrient starvation [22], macrophage infection [17] and by SDS stress [5]. Part of a genomic island [3]. Toxic in *E. coli* [23] but not in *M. smegmatis* [3]
Rv0627	Yes [1]	Structure determined in complex with C-terminal part of antitoxin (Rv0626) and biochemical evidence for ribonuclease activity [25]. Non-

		toxic when over-expressed in *E. coli* [23] or *M. smegmatis* [3] but toxic when over-expressed in *M. tuberculosis* [25]
Rv1953	No [1,18]	C-terminally truncated and lacking part of the PIN domain. Induced during adaptation to nutrient starvation [22]. Non-toxic in *E. coli* [23] and *M. smegmatis* [3]
Rv2010	No [1]	Induced during hypoxia [16] and in Balb/c mice [6], but repressed during nutrient starvation [11], adaptation to hypoxia [12] and in wild type H37Rv compared to a *phoP* mutant [13]. Protein identified in 30-d infected guinea pig lungs [26]. Part of a genomic island [3]. Non-toxic in *E. coli* [23] but toxic in *M. smegmatis* [3]
Rv2546	No [1]	Induced in Balb/c mice [6] and during treatment with SRI#967, a compound exhibiting strong anti-mycobacterial properties [27]. Non-toxic in *E. coli* [23] and *M. smegmatis* [3]
Rv2548	No [1]	Induced during hypoxia [16] and macrophage infections [17], but repressed in sputum [28]. Non-toxic in *E. coli* [23] but toxic in *M. smegmatis* [3]
Rv2549c	No [1]	Induced during macrophage infection [17] and in the presence of high iron concentrations [19]. Toxic in *E. coli* [23] but non-toxic in *M. smegmatis* [3]
Rv2829c	-	Induced during macrophage infection [3], hypoxia [3,12] and nutrient starvation [22]. Protein identified in 30-d infected guinea pig lungs [26]. Non-toxic in *E. coli* [23] but toxic in *M. smegmatis*[3]
Rv3320c	No [1]	Repressed during hypoxia [12] and nutrient starvation [11]. Part of a genomic island [3]. Non-toxic in *M. smegmatis* [3]
MSMEG_1284	-	Toxic in *M. smegmatis* [29]

a. Deduced from genome-wide essentiality screens

References

1. Sassetti CM, Boyd DH, Rubin EJ (2003) Genes required for mycobacterial growth defined by high density mutagenesis. Mol Microbiol 48: 77-84.
2. Stewart GR, Wernisch L, Stabler R, Mangan JA, Hinds J, et al. (2002) Dissection of the heat-shock response in *Mycobacterium tuberculosis* using mutants and microarrays. Microbiology 148: 3129-3138.
3. Ramage HR, Connolly LE, Cox JS (2009) Comprehensive functional analysis of *Mycobacterium tuberculosis* toxin-antitoxin systems: implications for pathogenesis, stress responses, and evolution. PLoS Genet 5: e1000767.
4. Provvedi R, Boldrin F, Falciani F, Palu G, Manganelli R (2009) Global transcriptional response to vancomycin in *Mycobacterium tuberculosis*. *Microbiology* 155: 1093-1102.
5. Manganelli R, Voskuil MI, Schoolnik GK, Smith I (2001) The *Mycobacterium tuberculosis* ECF sigma factor sigmaE: role in global gene expression and survival in macrophages. Mol Microbiol 41: 423-437.
6. Talaat AM, Lyons R, Howard ST, Johnston SA (2004) The temporal expression profile of *Mycobacterium tuberculosis* infection in mice. Proc Natl Acad Sci USA 101: 4602-4607.

7. Fontan P, Aris V, Ghanny S, Soteropoulos P, Smith I (2008) Global transcriptional profile of *Mycobacterium tuberculosis* during THP-1 human macrophage infection. Infect Immun 76: 717-725.
8. Miallau L, Faller M, Chiang J, Arbing M, Guo F, et al. (2009) Structure and proposed activity of a member of the VapBC family of toxin-antitoxin systems: VapBC-5 from *Mycobacterium tuberculosis*. J Biol Chem 284: 276-283.
9. Rifat D, Bishai WR, Karakousis PC (2009) Phosphate depletion: a novel trigger for *Mycobacterium tuberculosis* persistence. J Infect Dis 200: 1126-1135.
10. Dubnau E, Fontan P, Manganelli R, Soares-Appel S, Smith I (2002) *Mycobacterium tuberculosis* genes induced during infection of human macrophages. Infect Immun 70: 2787-2795.
11. Betts JC, Lukey PT, Robb LC, McAdam RA, Duncan K (2002) Evaluation of a nutrient starvation model of *Mycobacterium tuberculosis* persistence by gene and protein expression profiling. Mol Microbiol 43: 717-731.
12. Sherman DR, Voskuil M, Schnappinger D, Liao R, Harrell MI, et al. (2001) Regulation of the *Mycobacterium tuberculosis* hypoxic response gene encoding alpha -crystallin. Proc Natl Acad Sci USA 98: 7534-7539.
13. Walters SB, Dubnau E, Kolesnikova I, Laval F, Daffe M, et al. (2006) The *Mycobacterium tuberculosis* PhoPR two-component system regulates genes essential for virulence and complex lipid biosynthesis. Mol Microbiol 60: 312-330.
14. Fisher MA, Plikaytis BB, Shinnick TM (2002) Microarray analysis of the *Mycobacterium tuberculosis* transcriptional response to the acidic conditions found in phagosomes. J Bacteriol 184: 4025-4032.
15. Ioerger TR, Koo S, No EG, Chen X, Larsen MH, et al. (2009) Genome analysis of multi- and extensively-drug-resistant tuberculosis from KwaZulu-Natal, South Africa. PLoS One 4: e7778.
16. Muttucumaru DG, Roberts G, Hinds J, Stabler RA, Parish T (2004) Gene expression profile of *Mycobacterium tuberculosis* in a non-replicating state. Tuberculosis (Edinb) 84: 239-246.
17. Stewart GR, Patel J, Robertson BD, Rae A, Young DB (2005) Mycobacterial mutants with defective control of phagosomal acidification. PLoS Pathog 1: 269-278.
18. Lamichhane G, Zignol M, Blades NJ, Geiman DE, Dougherty A, et al. (2003) A postgenomic method for predicting essential genes at subsaturation levels of mutagenesis: application to *Mycobacterium tuberculosis*. Proc Natl Acad Sci U S A 100: 7213-7218.
19. Rodriguez GM, Voskuil MI, Gold B, Schoolnik GK, Smith I (2002) *ideR*, An essential gene in *Mycobacterium tuberculosis*: role of IdeR in iron-dependent gene expression, iron metabolism, and oxidative stress response. Infect Immun 70: 3371-3381.
20. Sherman DR, Voskuil M, Schnappinger D, Liao R, Harrell MI, et al. (2001) Regulation of the *Mycobacterium tuberculosis* hypoxic response gene encoding alpha -crystallin. Proc Natl Acad Sci U S A 98: 7534-7539.
21. Park HD, Guinn KM, Harrell MI, Liao R, Voskuil MI, et al. (2003) Rv3133c/dosR is a transcription factor that mediates the hypoxic response of *Mycobacterium tuberculosis*. Mol Microbiol 48: 833-843.
22. Hampshire T, Soneji S, Bacon J, James BW, Hinds J, et al. (2004) Stationary phase gene expression of *Mycobacterium tuberculosis* following a progressive nutrient depletion: a model for persistent organisms? Tuberculosis (Edinb) 84: 228-238.

23. Gupta A (2009) Killing activity and rescue function of genome-wide toxin-antitoxin loci of *Mycobacterium tuberculosis*. FEMS Microbiol Lett 290: 45-53.
24. Dutta NK, Mehra S, Didier PJ, Roy CJ, Doyle LA, et al. (2010) Genetic requirements for the survival of tubercle bacilli in primates. J Infect Dis 201: 1743-1752.
25. Miallau L, Faller M, Chiang J, Arbing M, Guo F, et al. (2009) Structure and proposed activity of a member of the VapBC family of toxin-antitoxin systems. VapBC-5 from *Mycobacterium tuberculosis*. J Biol Chem 284: 276-283.
26. Kruh NA, Troudt J, Izzo A, Prenni J, Dobos KM (2010) Portrait of a pathogen: The *Mycobacterium tuberculosis* proteome in vivo. PLoS One 5: e13938.
27. Waddell SJ, Stabler RA, Laing K, Kremer L, Reynolds RC, et al. (2004) The use of microarray analysis to determine the gene expression profiles of *Mycobacterium tuberculosis* in response to anti-bacterial compounds. Tuberculosis (Edinb) 84: 263-274.
28. Garton NJ, Waddell SJ, Sherratt AL, Lee SM, Smith RJ, et al. (2008) Cytological and transcript analyses reveal fat and lazy persister-like bacilli in tuberculous sputum. PLoS Med 5: e75.
29. Robson J, McKenzie JL, Cursons R, Cook GM, Arcus VL (2009) The *vapBC* operon from *Mycobacterium smegmatis* is an autoregulated toxin-antitoxin module that controls growth via inhibition of translation. J Mol Biol 390: 353-367.
