# Supplementary material for: VapC Toxins from Mycobacterium tuberculosis Are Ribonucleases that Differentially Inhibit Growth and Are Neutralized by Cognate VapB Antitoxins
Source: PLoS One. 2011 Jun 29;6(6):e21738. doi: 10.1371/journal.pone.0021738 (PMC3126847; doi:10.1371/journal.pone.0021738)
Supplement: Table S2 — VapC toxicity in M. smegmatis mc2155 assessed by transformation efficiency of VapC expression vector. (PDF) [file pone.0021738.s004.pdf]

Table S2. VapC toxicity in *M. smegmatis* mc^{2}155 assessed by transformation efficiency of VapC expression vector[a]

VapC	Vector	CFU/µg
-	pSE100	2.7×10^4
Rv0549c	pSE0549c	63
Rv0595c	pSE0595c	8
Rv0627	pSE0627	2.4×10^4
Rv1953	pSE1953	2.6×10^4
Rv2010	pSE2010	2.1×10^3
Rv2546	pSE2546	3.5×10^4
Rv2548	pSE2548	2.0×10^4
Rv2549c	pSE2549c	11
Rv2829c	pSE2829c	15
Rv3320c	pSE3320c	1
MSMEG_1284	pSESM1284	1.5×10^4

a. The data shown are from one of three independent experiments.
