# Supplementary material for: VapC Toxins from Mycobacterium tuberculosis Are Ribonucleases that Differentially Inhibit Growth and Are Neutralized by Cognate VapB Antitoxins
Source: PLoS One. 2011 Jun 29;6(6):e21738. doi: 10.1371/journal.pone.0021738 (PMC3126847; doi:10.1371/journal.pone.0021738)
Supplement: Table S3 — VapC toxicity in Mtb assessed by transformation efficiency of VapC expression vector. (PDF) [file pone.0021738.s005.pdf]

Table S3. VapC toxicity in Mtb assessed by transformation efficiency of VapC expression vector[a]

Wild type (H37Rv)			ΔRv2545-Rv2550c		
VapC	Vector	CFU/µg	VapC	Vector	CFU/µg
-	pSE100	3.0×10^{6}	-	pSE100	1.0×10^{5}
Rv0549c	pSE0549c	1.4×10^{4}	Rv0549c	pSE0549c	1.9×10^{5}
Rv0595c	pSE0595c	3.3×10^{3}	Rv0595c	pSE0595c	8.2×10^{2}
Rv0627	pSE0627	8.7×10^{6}	Rv0627	pSE0627	1.1×10^{6}
Rv1953	pSE1953	5.6×10^{6}	Rv1953	pSE1953	1.4×10^{5}
Rv2010	pSE2010	9.7×10^{6}	Rv2010	pSE2010	2.6×10^{6}
Rv2546	pSE2546	1.6×10^{6}	Rv2546	pSE2546	1.1×10^{5}
Rv2548	pSE2548	2.6×10^{6}	Rv2548	pSE2548	1.5×10^{4}
Rv2549c	pSE2549c	8.4×10^{2}	Rv2549c	pSE2549c	7.1×10^{2}
Rv2829c	pSE2829c	1.0×10^{4}	Rv2829c	pSE2829c	1.1×10^{3}
Rv3320c	pSE3320c	9.0×10^{2}	Rv3320c	pSE3320c	0

a. The data shown are from one of three independent experiments.
