# Supplementary material for: VapC Toxins from Mycobacterium tuberculosis Are Ribonucleases that Differentially Inhibit Growth and Are Neutralized by Cognate VapB Antitoxins
Source: PLoS One. 2011 Jun 29;6(6):e21738. doi: 10.1371/journal.pone.0021738 (PMC3126847; doi:10.1371/journal.pone.0021738)
Supplement: Table S4 — Strains and plasmids used in this study. (PDF) [file pone.0021738.s006.pdf]

Table S4. Strains and plasmids used in this study

Name	Description	Source
Strains		
M. tuberculosis		
H37Rv	ATCC 25618, virulent laboratory strain	Laboratory stock
H37Ra	ATCC25177, avirulent laboratory strain	Laboratory stock
Δ*Rv0595c*	Derivative of H37Rv carrying an unmarked deletion in the *vapC* gene *Rv0595c*	This work
Δ*Rv2009-Rv2010*	Derivative of H37Rv carrying an unmarked deletion in the *vapBC* operon *Rv2009-Rv2010*	This work
Δ*Rv2545-Rv2550c*	Derivative of H37Rv carrying an unmarked deletion spanning from *Rv2545* to *Rv2550c*	This work
M. smegmatis		
mc^2155	*ept-1*, efficient plasmid transformation mutant of mc^26	[1]
Δ*MSMEG1283-1284*	Derivative of mc^2155 carrying an unmarked deletion in the *vapBC* operon *MSMEG_1283-MSMEG_1284*	This work
mc^24517	*ept-1*, efficient plasmid transformation mutant of mc^26, expression strain with T7 RNA polymerase	[2]
Yeast		
AH109	*HIS3*, *ADE2*, *lacZ*, *MEL1* containing reporter strain for screening protein interactions, *MATa*, *trp1-901*, *leu2-3*, *112*, *ura3-52*, *his3-200*, *gal4_*, *gal80_*, *LYS2*::*GAL1*UAS-*GAL1*TATA-*HIS3*, *MEL1 GAL2*UAS*GAL2*TATA-*ADE2*, *URA3*::*MEL1*UAS-*MEL1*TATA-*lacZ*	Clontech
Plasmids		
p2NIL	*E. coli* cloning vector; Km^R	[3]
pGEM3Z(+)f	*E. coli* cloning vector; Ap^R	Promega
pGOAL17	Plasmid carrying the *lacZ* and *sacB* genes as a *PacI* cassette; Ap^R	[3]
pGOAL19	Plasmid carrying the *lacZ* , *sacB* and *hyg* genes as a *PacI* cassette; Ap^R	[3]

p2Δ0595cKO	Knockout vector for creating an unmarked deletion in *M. tuberculosis Rv0595c*; Km^R, Hyg^R	This work
p2Δ2009_10KO	Knockout vector for creating an unmarked deletion in *M. tuberculosis Rv2009-Rv2010*; Km^R, Hyg^R	This work
p2Δ2545_50cKO	Knockout vector for creating a *hyg*-marked deletion in *M. tuberculosis Rv2545-Rv2550c*; Km^R, Hyg^R	This work
p2ΔSM1283_84KO	Knockout vector for creating an unmarked deletion in *M. smegmatis MSMEG_1283-MSMEG_1284*; Km^R, Hyg^R	This work
pOLYG	*E. coli*-Mycobacterium shuttle vector; Hyg^R	[4]
pSE100	*E. coli*-Mycobacterium shuttle vector carrying P_{myc1}*tetO*; Hyg^R	[5]
pSE0549c	pSE100 carrying *Rv0549c* under control of P_{myc1}*tetO*; Hyg^R	This work
pSE0627	pSE100 carrying *Rv0627* under control of P_{myc1}*tetO*; Hyg^R	This work
pSE1953	pSE100 carrying *Rv1953* under control of P_{myc1}*tetO* ; Hyg^R	This work
pSE2010	pSE100 carrying *Rv2010* under control of P_{myc1}*tetO*; Hyg^R	This work
pSE2829c	pSE100 carrying *Rv2829c* under control of P_{myc1}*tetO* ; Hyg^R	This work
pSE2546	pSE100 carrying *Rv2546* under control of P_{myc1}*tetO*; Hyg^R	This work
pSE2548	pE100 carrying *Rv2548* under control of P_{myc1}*tetO*; Hyg^R	This work
pSE2549c	pSE100 carrying *Rv2549c* under control of P_{myc1}*tetO* ; Hyg^R	This work
pSE3320c	pSE100 carrying *Rv3320c* under control of P_{myc1}*tetO*; Hyg^R	This work
pSE0550c_49c	pSE100 carrying *Rv0550c-Rv0549c* under control of P_{myc1}*tetO*; Hyg^R	This work
pSE0596c_95c	pSE100 carrying *Rv0596c-Rv0595c* under control of P_{myc1}*tetO*; Hyg^R	This work
pSE2550c_49c	pSE100 carrying *Rv2550c-Rv2549c* under control of P_{myc1}*tetO*; Hyg^R	This work
pSE2830c_29c	pSE100 carrying *Rv2830c-Rv2829c* under control of P_{myc1}*tetO*; Hyg^R	This work
pSESM1284	pSE100 carrying *MSMEG_1284* under control of P_{myc1}*tetO*; Hyg^R	This work
pSE2546_FLAG	pSE100 carrying a C-terminally FLAG-tagged *Rv2546* under control of P_{myc1}*tetO*; Hyg^R	This work
pSE2549c_FLAG	pSE100 carrying a C-terminally FLAG-tagged *Rv2549c* under control of P_{myc1}*tetO*; Hyg^R	This work
pMC1s	L5-based integration vector carrying P_{smyc}-*tetR*; Km^R	[5]

pMC1r	Derivative of pMC1s; Km^R	This work
pMC2m	L5-based integration vector carrying P_{imyc}-*tetR*; Km^R	D. Schnappinger & S. Ehrt
pTTP1B	Tweety-based integration vector; Ap^R; Km^R	[6]
pTTP1BG	Derivative of pTTP1B with Km^R marker replaced by Gm^R marker from pML10 [7]; Gm^R	This work
pTTP1BGs	Derivative of pTTP1BG carrying P_{smyc}-*tetR* from pMC1s; Gm^R	This work
pTT2549c	pTTP1BGs derivative carrying P_{myc1}*tetO*::*Rv2549c*; Gm^R	This work
pTT0595c	pTTP1BGs derivative carrying P_{myc1}*tetO*::*Rv0595c*; Gm^R	This work
pOLYGaa	pOLYG derivative carrying *M. smegmatis* acetamidase promoter (P_{ami}) from pAGAN11 [8]; Hyg^R	D. Warner
pMAP	Derivative of pMC1r carrying P_{ami} cloned as a 4.4-kb *Pvu*II/*Cla*I fragment from pOLYGaa; Km^R	This work
pMAP2550c	Integration vector carrying P_{ami}::*Rv2550c*; Km^R	This work
pMAP2830c	Integration vector carrying P_{ami}::*Rv2830c*; Km^R	This work
pMAP0596c	Integration vector carrying P_{ami}::*Rv0596c*; Km^R	This work
pGADT7	Y2H vector to produce AD fusions, *GAL4*(768-881)AD, *LEU2*, HA epitope tag; Ap^R	Clontech
pGAD0595c	pGADT7 containing *Rv0595c* fused to *GAL4* AD	This work
pGAD0596c	pGADT7 containing *Rv0596c* fused to *GAL4* AD	This work
pGAD2549c	pGADT7 containing *Rv2549c* fused to *GAL4* AD	This work
pGAD2550c	pGADT7 containing *Rv2550c* fused to *GAL4* AD	This work
pGAD2830c	pGADT7 containing *Rv2830c* fused to *GAL4* AD	This work
pGBKT7	Y2H vector to produce BD fusions, *GAL4*(1-147)DNA-BD, *TRP1*, c-MYC epitope tag; Km^R	Clontech
pGBK0595c	pGBKT7 containing *Rv0595c* fused to *GAL4* BD	This work
pGBK0596c	pGBKT7 containing *Rv0596c* fused to *GAL4* BD	This work
pGBK2549c	pGBKT7 containing *Rv2549c* fused to *GAL4* BD	This work
pYUB28b	*E. coli*-Mycobacterium shuttle vector with T7 promoter and C-terminal His-tag; Hyg^R	[9]
pYUBRv0065A-5	pYUB28b derivative carrying the Rv0065A-Rv0065 operon for protein expression; Hyg^R	This work
pYUBRv0616A-6	pYUB28b derivative carrying the Rv0616A-Rv0617 operon for protein expression; Hyg^R	This work

References

1. Snapper SB, Melton RE, Mustafa S, Kieser T, Jacobs WR, Jr. (1990) Isolation and characterization of efficient plasmid transformation mutants of *Mycobacterium smegmatis*. Mol Microbiol 4: 1911-1919.
2. Wang F, Jain P, Gulten G, Liu Z, Feng Y, et al. (2010) *Mycobacterium tuberculosis* dihydrofolate reductase is not a target relevant to the antitubercular activity of isoniazid. Antimicrob Agents Chemother 54: 3776-3782.
3. Parish T, Stoker NG (2000) Use of a flexible cassette method to generate a double unmarked *Mycobacterium tuberculosis tlyA plcABC* mutant by gene replacement. Microbiology 146 1969-1975.
4. O'Gaora P, Barnin S, Hayward C, Filley E, Rook G, et al. (1997) Mycobacteria as immunogens: development of expression vectors for use in multiple mycobacterial species. Med Princ Prac 6: 91–96.
5. Ehrt S, Guo XV, Hickey CM, Ryou M, Monteleone M, et al. (2005) Controlling gene expression in mycobacteria with anhydrotetracycline and Tet repressor. Nucleic Acids Res 33: e21.
6. Pham TT, Jacobs-Sera D, Pedulla ML, Hendrix RW, Hatfull GF (2007) Comparative genomic analysis of mycobacteriophage Tweety: evolutionary insights and construction of compatible site-specific integration vectors for mycobacteria. Microbiology 153: 2711-2723.
7. Labes M, Puhler A, Simon R (1990) A new family of RSF1010-derived expression and lac-fusion broad-host-range vectors for gram-negative bacteria. Gene 89: 37-46.
8. Parish T, Mahenthiralingam E, Draper P, Davis EO, Colston MJ (1997) Regulation of the inducible acetamidase gene of *Mycobacterium smegmatis*. Microbiology 143 (Pt 7): 2267-2276.
9. Bashiri G, Rehan AM, Greenwood DR, Dickson JM, Baker EN (2010) Metabolic engineering of cofactor F420 production in *Mycobacterium smegmatis*. PLoS One 5: e15803.
