# Supplementary material for: VapC Toxins from Mycobacterium tuberculosis Are Ribonucleases that Differentially Inhibit Growth and Are Neutralized by Cognate VapB Antitoxins
Source: PLoS One. 2011 Jun 29;6(6):e21738. doi: 10.1371/journal.pone.0021738 (PMC3126847; doi:10.1371/journal.pone.0021738)
Supplement: Table S5 — Oligonucleotides used to create knockout vectors for allelic exchange mutagenesis. (PDF) [file pone.0021738.s007.pdf]

Table S5. Oligonucleotides used to create knockout vectors for allelic exchange mutagenesis

Gene(s)	Internal deletion size (bp)	Knockout vector	Oligonucleotide pairs used for vector construction	
			**Sequence (5'-3')[a]**	**Region amplified**
M. tuberculosis				
Rv0595c	325	p2Δ0595cKO	GCAGAAGCTTACGAAAAGATCGGCGTC<br>GGCTAGATCTGTACGTTCACCACCGCAC	1990 bp amplicon containing 10 bp of the 5' end of Rv0595c and 1980 bp of upstream sequence
			GAGCAGATCTGACCCAAGACAACGACTA<br>GTATGCGGCCGCCTAACCTTCACCAGAC	2027 bp amplicon containing 58 bp of the 3' end of Rv0595c and 1969 bp of downstream sequence
Rv2545-Rv2550c	2415	p2Δ2545_50cKO	TGATAAGCTTGATGACGATCTCGCGCAG<br>TAATAGATCTGAGATATATGCATTGGA	2052 bp amplicon containing 131 bp of the 5' end of Rv2545 and 1921 bp of upstream sequence
			ATGTAGATCTGCAGCCTTTTCACA<br>TTATGGTACCAGGGACTATCAG	1776 bp amplicon containing 42 bp of the 3' end of Rv2550c and 1734 bp of downstream sequence
M. smegmatis				
MSMEG_1283-MSMEG_1284	378	p2ΔSM1283_84KO	TGATAAGCTTGCTCATACGGCCAGGC<br>TAATAGATCTCTGGCCAGCCGGTCGG	965 bp amplicon containing 23 bp of the 5' end of MSMEG1283 and 932 bp of upstream sequence
			CGCCGAGATCTGTGGTTGCATCGC<br>TGATGGTACCGAGCAGTGGCTACTGG	1099 bp amplicon containing 219 bp of the 3' end of MSMEG1284 and 880 bp of downstream sequence

a. Restriction sites used for cloning are underlined
