# Supplementary material for: VapC Toxins from Mycobacterium tuberculosis Are Ribonucleases that Differentially Inhibit Growth and Are Neutralized by Cognate VapB Antitoxins
Source: PLoS One. 2011 Jun 29;6(6):e21738. doi: 10.1371/journal.pone.0021738 (PMC3126847; doi:10.1371/journal.pone.0021738)
Supplement: Table S6 — Oligonucleotides used for expression vector construction, yeast two-hybrid (Y2H) vector construction and RT-PCR. (PDF) [file pone.0021738.s008.pdf]

Table S6. Oligonucleotides used for expression vector construction, yeast two-hybrid (Y2H) vector construction and RT-PCR

**Name**	**Forward (5'-3')[a]**	**Reverse (5'-3')**
**Oligonucleotides used for expression vector construction**		
Toxins and toxin-antitoxin modules under control of P_{myc1}*tetO* in pSE100		
Rv0549c	TAATGGATCCGGAAGGAATCGAAGCGTGAGAGCATC	CGCACTGCAGTGTCGCTCGGCAATG
Rv0550c-Rv0549c	ATCTGGATCCGGAGGAAGGCCTTGTTGAGCCG	CGCACTGCAGTGTCGCTCGGCAATG
Rv0595c	ATGTGGATCCAGGAAGGACGATGTGCGGTGGTG	TACTCTGCAGCCGGCGCGATCAGGTA
Rv0596c-Rv0595c	CTAAGGATCCGGAGGAACTCGTAGCATGTCTGCT	TACTCTGCAGCCGGCGCGATCAGGTA
Rv0627	TAGTCTGCAGGAAGGATCCGGTGAGCACGACG	TACTAAGCTTCGTCGACTGAGGCCGC
Rv1953	TACTGGATCCGGAAGGACGCAACTGTGACCTACGT	GAATCTGCAGTGTACAGTCGCAACCTG
Rv2010	TACTGGATCCGGAAGAGTCGTAGATGATCGTC	TGCACTGCAGATGTGAGACACGAAATC
Rv2546	ACTGGGATCCGGAAGGTGATGGTGTTCTGCGTC	TGATAAGCTTGGGTCACCTGAGTCCGCATG
Rv2546_FLAG	ACTGGGATCCGGAAGGTGATGGTGTTCTGCGTC	GGGGAAG*CTTCTACTTGTCATCGTCATCCTTGTAATCTATATCGTGATCTTTATAATCACCGTCATGGTCTTTGTAGTC*AAGGGTCCCGCGCGGCAC
Rv2548	ACTGGGATCCGGAAGGTCTGGCGTGAAGCTGAT	CGCGAAGCTTGCTGATGCCCCAGGGAGT
Rv2549c	TGATGGATCCGGAAAGCACGAATGATCTTC	TGTAAAGCTTCAACGCAACGCAGCCCTGT
Rv2549c_FLAG	TGATGGATCCGGAAAGCACGAATGATCTTC	GGGGAAG*CTTCTACTTGTCATCGTCATCCTTGTAATCTATATCGTGATCTTTATAATCACCGTCATGGTCTTTGTAGTC*CTCGGGTCTTACCTCGAC
Rv2550c-Rv2549c	CGTAGGATCCGGAGGAACAGCATTATGCTAGTGG	TGTAAAGCTTCAACGCAACGCAGCCCTGT
Rv2829c	GGGCGGATCCGGAAGGAACGTTTCATGACGACGGTG	GCGGCTGCAGTTCGGTGATGTTGCGGC

Rv2830c-Rv2829c	CTGAGGATCCAGAAGAAAAATGACCGCTACGGA	GCGGCTGCAGTTCGGTGATGTTGCGGC
Rv3320c	ATAGGATCCAGGAAGGAGGCCGTGTGATCGA	TCCTCTGCAGCCGGGTCTACAGCGTT
MSMEG_1284	TTCTAAGCTTGGAAGGTCCTGATGGTTATCGAC	CGATGGATCCTGACCTGAATTCTGACCT
Antitoxins under control of P_{ami} in Pmap		
Rv2550c	CGTAGAATTCGGAGGAACAGCATTATGCTAGTGG	GCCCATCGATTAAAGTGCAGCCCAGAA
Rv0596c	TAGTGAATTCGTGAGGAATCGTAGCATGTCTGCTA	TCACATCGATTCATACGTTCACCACCGCACA
Rv2830c	TACTGAATTCGTGAGGAAAGAAAAATGACCGCTACG	TGCAATCGATACTATGTCATGAAACGTTCCACG
Toxin-antitoxin modules under control of T7 in pYUB28b		
Rv0065A-5	GTTACCATGGCTACCATTCAAGTTCGGGA	GCAAGCTTCCGAACGAGTTTGATTTCG
Rv0616A-5	TAATCCATGGTGCGCACTACCATCGA	TAAAGCTTGGTGGTCGTTGGAATGAG
**Oligonucleotides used for Y2H vector construction**		
Rv0595c	TGACATATGAACGTACGGCGCGCC	TGAGAATTCTCAGATCGTTATGACCTC
Rv0596c	TAACATATGTCTGCTACGATACCC	TGAGAATTCTCACCACCGCACATCGTC
Rv2549c	TAACATATGATCTTCGTCGACACG	TGAGAATTCCTACTCGGGTCTTACCTC
Rv2550c	GGCCATATGCTAGTGGCGTATATT	GGCGAATTCTCATTCGTGCTTTCCGTA
Rv2830c	TAACATATGACCGCTACGGAGGTG	TGAGAATTCTCATGAAACGTTCCACGA
**Oligonucleotides used for RT-PCR**		
Rv0549c	TGCCATGGTGGATCTACTGGCTCGC	AACACCTCTGCATCGAAGTGCGCCG
Rv0595c	AACCACTAGGCATCGACGAGGCGG	CGTTGATCGGCACGCGCAGTTTGG
Rv0627	GGTGTTCATCGCGACCGAAAGCGG	GATTCCAGGGTCGCCAGGCGTTGA
Rv2010	ACGTCAGAGTCGTTGGCCAGTCGC	GCTCCATCATCACCACCTCGGGCA

Rv2546	TATACCAGTCGGTGCCGAAA	ACACGATGGTGCCTGAAAGT
Rv2549c	TCTTCCAGGTCGGCTGTTAC	GATGACCTCCAACCATGTCC
Rv2829c	CTCGACTCGCATGTGGCCTA	AGCCAGCTCGAACCACGAA
M. smegmatis sigA	GGGCGTGATGTCCATCTCCT	GTATCCCGGTGCACATGGTC

a. Restriction sites used for cloning are underlined

b. Italicized sequence represents the 3× FLAG tag (Asp Tyr Lys Asp His Asp Gly Asp Tyr Lys Asp His Asp Ile Asp Tyr Lys Asp Asp Asp Asp Lys)
